# Ginkgotides: Proline-Rich Hevein-Like Peptides from Gymnosperm *Ginkgo biloba*

**DOI:** 10.3389/fpls.2016.01639

**Published:** 2016-11-03

**Authors:** Ka H. Wong, Wei Liang Tan, Aida Serra, Tianshu Xiao, Siu Kwan Sze, Daiwen Yang, James P. Tam

**Affiliations:** ^1^School of Biological Sciences, Nanyang Technological UniversitySingapore, Singapore; ^2^Department of Biological Sciences, National University of SingaporeSingapore, Singapore

**Keywords:** *Ginkgo biloba*, hevein, hevein-like peptides, proline-rich, anti-fungal, cysteine-rich peptides

## Abstract

Hevein and hevein-like peptides belong to the family of chitin-binding cysteine-rich peptides. They are classified into three subfamilies, the prototypic 8C- and the 6C- and 10C-hevein-like peptides. Thus far, only five 8C-hevein-like peptides have been characterized from three angiosperms and none from gymnosperm. To determine their occurrence and distribution in the gymnosperm, *Ginkgo biloba* leaves were examined. Here, we report the discovery and characterization of 11 novel 8C-hevein-like peptides, namely ginkgotides gB1–gB11. Proteomic analysis showed that the ginkgotides contain 41–44 amino acids (aa), a chitin-binding domain and are Pro-rich, a distinguishing feature that differs from other hevein-like peptides. Solution NMR structure determination revealed that gB5 contains a three β-stranded structure shaped by a cystine knot with an additional disulfide bond at the C-terminus. Transcriptomic analysis showed that the ginkgotide precursors contain a three-domain architecture, comprised of a C-terminal tail (20 aa) that is significantly shorter than those of other 8C- and 10C-hevein-like peptides, which generally contain a protein cargo such as a Barwin-like protein (126 aa) or class I chitinase (254 aa). Transcriptomic data mining found an additional 48 ginkgotide homologs in 39 different gymnosperms. Phylogenetic analysis revealed that ginkgotides and their homologs belong to a new class of 8C-hevein-like peptides. Stability studies showed that ginkgotides are highly resistant to thermal, acidic and endopeptidase degradation. Ginkgotides flanked at both the N- and C-terminal ends by Pro were resistant to exopeptidase degradation by carboxypeptidase A and aminopeptidase. Antifungal assays showed that ginkgotides inhibit the hyphal growth of phyto-pathogenic fungi. Taken together, ginkgotides represent the first suite of hevein-like peptides isolated and characterized from gymnosperms. As a group, they represent a novel class of 8C-hevein-like peptides that are Pro-rich and protein-cargo free. Our findings also suggest that the ginkgotide scaffold could be useful for engineering metabolic-stable peptide therapeutics.

## Introduction

**FIGURE 1:**
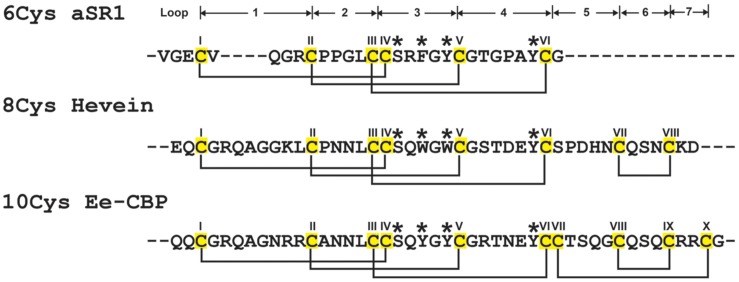
**Summary of hevein-like peptide subfamilies.** The backbone segments between adjacent Cys (loop) are labeled as 1–7. The chitin-binding domain, located at loops 3 and 4, are conversed among hevein-like peptides, which consisted of a Ser and three aromatic amino acids (marked with ^∗^). The 6C-hevein-like peptide (aSR1) is a truncated version of 8C-hevein-like peptide (hevein) with the loops 5–7 (CysVII and CysVIII) are omitted, whereas 10C-hevein-like peptide (Ee-CBP) have an additional disulfide bond between C-terminus and loop 5. The eight Cys are highlighted in yellow.

Plants have evolved complex and effective defense mechanisms to ward off pathogens and pests (Hegedus and Marx, 2013). General pathogen resistance is accomplished by reinforcement of cell walls, alternating cell membrane permeability and the release of pathogenesis-related biomolecules, hydrolytic enzymes and secondary metabolites (Sels et al., 2008; Wong et al., 2011; Ponce de Leon and Montesano, 2013). Among the pathogenesis-related biomolecules, cysteine-rich peptides (CRPs) are known to play an important role in the plant defense against fungal pathogenic attacks (Cammue et al., 1994; Sels et al., 2008; Hegedus and Marx, 2013; Ponce de Leon and Montesano, 2013). Hevein, a cystine knot CRP and the prototype of the hevein and hevein-like peptide family, was first isolated from the latex of the rubber tree *Hevea brasiliensis* in Archer (1960). Since then, 20 hevein-like peptides from 10 different angiosperms have been isolated and characterized (Hammami et al., 2009). Hevein and hevein-like peptides consisted of 29–45 amino acids (aa) and are both Cys- and Gly-rich. In addition, these peptides contain a conserved chitin-binding domain, which gives them the ability to bind to chitin, a polymer of β-1,4-*N*-acetyl-D-glucosamine and the building block of fungal cell walls and exoskeletons of insects and arthropods (Rinaudo, 2006). The chitin-binding domain consists of one Ser and two aromatic amino acid residues located at the intercysteine loop 3 and one aromatic residue at loop 4 (**Figure 1**). This conserved chitin-binding domain can also be found in class I chitinases, such as Cht-2 from rice *Oryza sativa* (Takakura et al., 2000) and urtica dioica agglutinin (UDA) from the stinging nettle *Urtica dioica* (Van Damme et al., 1988).

The family of hevein and hevein-like peptides can be divided into three subfamilies based on the number of Cys residues present in their sequences (**Figure 1**). The prototypic subfamily is the 8C-heveins, which with three disulfide pairs comprising a cystine knot at the N-terminus and the fourth disulfide bond at the C-terminus. The other two subfamilies, 6C- and 10C-hevein-like peptides, contain 6 and 10 cysteine residues, respectively. They share a similar cystine knot motif and a chitin-binding domain with the 8C-hevein-like peptides subfamily (Tam et al., 2015). However, the 6C-hevein-like peptides was considered as a truncated version of the 8C-hevein-like peptides with the deletion of the fourth disulfide pair at the C-terminus (intercysteine loops 5 and 6) (Broekaert et al., 1997; Zasloff, 2002; Tam et al., 2015). In contrast, the 10C-hevein-like peptides contain an additional disulfide bond, which is either located entirely at the C-terminus or found as a cross-link between the C-terminus and one of the loops formed by a cystine knot. For example, the additional disulfide bond of the 10C-hevein-like peptide found in the spindle tree *Euonymus europaeus* Ee-CBP is located between the C-terminus and loop 5 (Van den Bergh et al., 2002a,b), whereas, the additional disulfide bond of the 10C-hevein-like peptide in *Triticum kiharae* WAMP-1a is located between the C-terminus and loop 2 (Odintsova et al., 2009).

*Ginkgo biloba*, which belongs to the family Ginkgoaceae, is one of the oldest species of gymnosperm dates back to the late Jurassic period for >150 million years (vanBeek, 2003; Hori et al., 2012). *G. biloba* leaves have been widely used in traditional Chinese medicine to improve blood circulation, relieve pain and reduce cholesterol levels (China Pharmacopoeia Commission, 2010). The major bioactive chemical constituents in *G. biloba* leaves include ginkgolides, bilobalides, polyprenols, carotenoids, and polyphenols (Hong Kong Chinese Materia Medica Standards, 2010). *G. biloba* leaves products are one of the best-selling nutraceutics around the world for treating dementia, vertigo and dizziness, improving mental function and relieving anxiety (McKenna et al., 2001; Al-Achi, 2008; Bent, 2008; Zhang et al., 2008).

The presence of a cystine knot endows heveins and hevein-like peptides with high tolerance to acidic, thermal and enzymatic degradation. This chemical stability is important for CRPs to be relevant as bioactive compounds in traditional medicines, which are generally prepared as decoctions and taken orally. However, little is known about the therapeutic potential of the hevein-like peptide family in medicinal herbs, an area which is highly under-explored and is our current research interest (Nguyen et al., 2011a, 2014; Tam et al., 2015). With an increasing interest in *G. biloba* worldwide as nutraceuticals, there is a need to study the putative active CRPs in these products.

Herein, we report the discovery and characterization of 11 8C-hevein-like peptides, named ginkgotides and abbreviated gB1 to gB11, from *G. biloba* leaves. Using a combination of proteomic, transcriptomic and bioinformatic analyses, we show that these peptides are distinguished from other hevein-like peptides by their Pro-rich and their C-terminus protein-cargo-free nature. Transcriptomic data mining revealed an additional 42 ginkgotide homologs, which expanded the library to 58 members. Together, these peptides represent a new class in the subfamily of 8C-hevein-like peptides and are primarily distributed in gymnosperms. This finding is in agreement with the taxonomical classification of ginkgotide-containing plants in gymnosperms (Chaw et al., 1997). Taken together, our findings not only confirm the presence of hevein-like peptides in gymnosperms but also suggest that hevein-like peptides have more diverse sequences than previously thought. To our knowledge, ginkgotides represent the first suite of hevein-like peptides isolated from gymnosperms. Their discovery furthers our understanding regarding the occurrence and distribution of hevein-like peptides *in planta* and could provide insights into the evolution of defense mechanism in modern gymnosperms.

## Materials and Methods

### General Experimental Procedures

High-performance liquid chromatography (HPLC) and ultra-performance liquid chromatography (UPLC) analyses were performed on a Prominence UFLC and a Nexera UHPLC system (Shimadzu, Kyoto, Japan), respectively. The detection wavelength was set at 220 nm. A Aeris peptide XB-C_18_ column (Phenomenex, Torrance, CA, USA; particle size 5 μm, 250 mm × 22 mm) was used for preparative reversed-phase HPLC (RP-HPLC). A polysulfoethyl A column (PolyLC, Boston, MA, USA) was used for the strong cation exchange HPLC (SCX-HPLC). Mass spectrometry analysis of the crude extracts and HPLC fractions were carried out on an ABI 4800 MALDI-TOF/TOF system (Applied Biosystem, Waltham, MA, USA). Absorbance in anti-fungal and cytotoxicity assays was measured using an Infinite 200 PRO Tecan microplate reader (Tecan, Männedorf, Switzerland). Chemical reagents and solvents used in this study were of analytical grade and purchased from Sigma-Aldrich (St. Louis, MO, USA) unless otherwise stated.

### Plant Materials

Dried *G. biloba* leaves were purchased from Hung Soon Medical Trading, Ltd, Singapore. The sample was authenticated by an experienced traditional Chinese medicine practitioner, Dr. Zhao Yan, based on the macroscopic and microscopic characteristics described in China Pharmacopoeia Commission (2010), Wong et al. (2013, 2014, 2015a,b). A voucher of each sample was deposited at the Nanyang Technological University Herbarium, School of Biological Sciences, Singapore.

### Isolation and Purification of Ginkgotides

Dried *G. biloba* leaves (2 kg) were homogenized in 20 L of sodium acetate-acetic acid buffer at pH 3.7 with 1 mM phenylmethanesulfonyl fluoride and incubated for 12 h at 4°C. The homogenate was squeezed through a layer of cheesecloth and the filtrate was centrifuged at 9000 rpm at 4°C for 20 min. The supernatant was loaded onto a reversed-phase flash column with 500 g of C_18_ powder (Havre de Grace, MD, USA) packed in a Büchner funnel (250 mm × 22 mm). Elution was carried out using increasing concentrations of ethanol (20, 40, 60, and 80% v/v). Eluents with the desired peptides were pooled and loaded onto a Sepharose Fast Flow SP (GE Healthcare Life Sciences, Little Chalfont, UK) SCX flash column. The column was percolated with 20 mM potassium dihydrogen phosphate at pH 3.0 until the unbound peptides were completely removed. Bound peptides were eluted by adding 20 mM potassium dihydrogen phosphate with 1 M sodium chloride at pH 3.0. To obtain purified peptides, multiple rounds of preparative RP- and SCX-HPLC were performed. In preparative RP-HPLC, a gradient elution at a flow rate of 5 mL/min was employed using buffer A [0.1% trifluoroacetic acid (TFA) in deionized water] and buffer B (0.1% TFA in acetonitrile) as follows: 20–20% B from 0.01–20 min, 20–25% B from 20–100 min, 25–40% B from 100–110 min, and 40–100% B from 110–120 min. For preparative SCX-HPLC, a gradient method at a flow rate of 5 mL/min was employed using buffer A (20 mM potassium dihydrogen phosphate at pH 3) and buffer B (20 mM potassium dihydrogen phosphate and 1 M sodium chloride at pH 3) as follows: 0–100% B from 0.01–60 min and 100–100% B from 60–90 min.

### Ginkgotide Sequence Determination

Reduction and alkylation of ginkgotides was performed as previously described (Nguyen et al., 2012, 2013). Lyophilized ginkgotides (10 μg) were dissolved in 30 μL of 20 mM dithiothreitol (DTT) and incubated at 37°C for 1 h. The reduced ginkgotides were alkylated with 200 mM iodoacetic acid (IAA) at 37°C for 1 h in the dark. The reduced and alkylated sample was desalted using a C_18_ Zip-tip and dried by SpeedVac at room temperature. Prior to mass spectrometry analysis, the peptides were re-dissolved in 0.1% formic acid (FA). LC–MS/MS analysis were performed using a Dionex UltiMate 3000 UHPLC system coupled with an Orbitrap Elite mass spectrometer (Thermo Fisher Scientific, Inc., Bremen, Germany). The mobile phase was 0.1% FA as eluent A and 90% ACN 0.1% FA as eluent B, with a flow rate of 0.3 μL/min. Peptide separation was performed with a 60 min gradient as follows: 3% of mobile phase B for 1 min, 3–35% of mobile phase B over 47 min, 35–50% of mobile phase B over 4 min, 50–80% of mobile phase B over 0.1 min and 80% of mobile phase B for 1.3 min followed by reversion to the initial conditions for 0.1 min and isocratic maintenance for 6.5 min.

The mass spectrometer was set to positive mode for data acquisition using the LTQ Tune Plus software (Thermo Fisher Scientific, Bremen, Germany). The spray was generated using a Michrom’s Thermo CaptiveSpray nanoelectrospray ion source (Bruker-Michrom, Auburn, CA, USA). The data were acquired by alternating the Full FT-MS (350–2000 m/z, resolution 60.000, with 1 μscan per spectrum) with an FT-MS/MS scan applying 27, 30, and 32% normalized collision energy in high-energy collisional dissociation (110–2000 m/z, resolution 30.000, with 2 μscan averaged per MS/MS spectrum) where the three most intense ions with charge >2+ were isolated with a 3 Da mass isolation window and fragmented. The capillary temperature was set at 250°C with a source voltage of 1.5 kV. The automatic gain control for full scan-MS and MS/MS was set to 1 × 106. Data from the LC–MS/MS analysis was processed using PEAKS studio version 7.5 [50] (Bioinformatics Solutions, Waterloo, ON, Canada). A parent error tolerance of 10 ppm and a fragment error tolerance of 0.05 Da were applied.

### Chitin-Binding Assay

This assay was performed as previously described (Kini et al., 2015). Purified and alkylated ginkgotides (40 μM) were mixed with 60 μL of chitin beads (BioLabs, UK) in chitin binding buffer [140 mM NaCl, 10 mM Tris, 1 mM EDTA and 0.1% (v/v) Tween at pH 8.0] and incubated at room temperature for 4 h. After incubation, the mixture was centrifuged at 10,000 *g* for 10 min, and the supernatant was removed. The chitin beads were then washed with chitin binding buffer. Chitin-bound peptides were eluted by adding 500 mM acetic acid at pH 3.0. The supernatants and eluents were analyzed using UPLC and MALDI-TOF MS.

### Heat and Acid Stability Assay

Purified ginkgotides (200 μM) were incubated in a water bath at 100°C or 1 M hydrochloride acid (pH 2) for 2 h. At each time-point (0 and 1 h), 20 μL of the treated sample was aliquot and quenched in an ice bath for 10 min or by adding 20 μL of 0.2 M sodium hydroxide. RP-UPLC was performed to determine the amounts of ginkgotide present before and after treatment.

### Endoproteolytic Enzyme Stability Assay

Purified ginkgotides (200 μM) were added to 100 μL of 100 mM ammonium bicarbonate buffer (pH 7.8) and incubated in a water bath at 37°C for 6 h. At each time-point (0 and 6 h), 20 μL of the treated sample was aliquoted and quenched by adding 5 μL of 1 M hydrochloric acid. RP-UPLC was performed to determine the amounts of ginkgotide present before and after treatment.

### Exoproteolytic Enzyme Stability Assay

Purified ginkgotides (200 μM) were added to 50 mM Tris-HCl, 100 mM sodium chloride in 100 nM carboxypeptidase A or 20 mM tricine and 0.05% bovine serum albumin (pH 8.0) in 20 U/mL aminopeptidase I. The mixture was incubated in a water bath at 37°C for 4 h. At each time-point (0 and 6 h), 20 μL of the treated sample was aliquoted and quenched by adding 5 μL of 1 M hydrochloric acid. RP-UPLC was performed to determine the amounts of ginkgotide present before and after treatment.

### NMR Structure Determination

The sequential assignment was determined using two-dimension total correlation spectroscopy and nuclear overhauser spectroscopy (2D TOCSY and NOESY). The NMR experiments were conducted on a 800 MHz NMR spectrometer (Bruker, Chicago, IL, USA) with a cryogenic probe. The temperature of the NMR experiment was set at 25°C. The protein concentration of the gB5 sample was 1 mM, containing 5% D_2_O and 95% H_2_O (pH 3.5). For ^1^H,^1^H-2D TOCSY and NOESY, the mixing times were 80 and 200 ms, respectively. The carrier frequency of ^1^H was at 4.745 ppm. The spectrum width was 12 ppm. The H/D exchange NMR experiment was conducted at 25°C using a Bruker 600 MHz NMR spectrometer (Bruker, Chicago, IL, USA) equipped with a cryogenic probe. The peptide sample was first lyophilized, and 1D ^1^H NMR spectra were recorded immediately after the sample was dissolved in D_2_O. In total, 20 1D spectra were recorded at varied time intervals within 4 h. The spectra were processed using NMRPipe software (Delaglio et al., 1995). The NOE cross-peaks were assigned using Sparky software based on the 2D NOESY and TOCSY experiment (Goddard and Kneller). The structure calculation was performed using CNSsolve 1.3 software (Brunger et al., 1998). The distance restraints were divided into three classes based on the intensities of NOE peaks: strong, 0 < d ≤ 1.8 Å; medium, 1.8 < d ≤ 3.4 Å; and weak, 3.4 < d ≤ 5 Å. There were six hydrogen bonds used in the structure calculation based on the H/D exchange experiment. The distance between HN and O was defined as 2.2-0.6 Å, and the distance between N and O was defined as 3.3-0.8 Å. The structure was verified using the PROCHECK program^1^ and displayed using Chimera version 1.6.2 (Huang et al., 1996) or Pymol version 1.8 (Schröginger, 2015).

### Anti-fungal Assay

The anti-fungal activities of the ginkgotides were examined using a radial disk diffusion assay as previously described (Ye and Ng, 2002). Five common phyto-pathogenic fungal strains were acquired from the China Center of Industrial Culture Collection (Beijing, China), including *Curvularia lunata* (CICC 40301), *Fusarium oxysporum* (CICC 2532), *Bipolaris maydis* (CICC 2530), *Verticillium dahlia* (CICC 2534), and *Rhizoctonia solani* (CICC 40259). The fungal strains were maintained in 90 mm × 15 mm Petri dishes containing 20 mL of potato dextrose agar. Fungal mycelia were harvested by punching a hole from the actively growing fungal plate and transferring the material to the center of a new agar plate. The plate was incubated at 25°C for 48 h to allow the formation of a radical mycelia colony. After that, four round filter papers (0.65 cm in diameter) were placed equidistant of 1 cm away from the rim of the mycelia colony. Aliquots of 17.5, 35, and 70 μg of ginkgotide were diluted in 20 μL of deionized water and added to the respective filter paper, and 20 μL of deionized water was used as a negative control. The plates were incubated at 25°C for 48 h until the mycelia colony had enveloped half of the surface of the filter paper disk with deionized water alone (negative control). The peptide was considered to possess anti-fungal activity if a crescent-shaped zone was observed around the disk.

The half maximal inhibitory concentration levels (IC_50_) were determined as described by Wiegand et al. (2008). The spores were harvested from a 5-day old actively growing fungal plate and suspended in 5 mL of half-strength potato dextrose broth. In a 96-well microplate, 80 μL of the spore solution (3000 spores/mL) was mixed with 20 μL of ginkgotides at different concentrations. The plates were incubated at 25°C for 24 h, and the viabilities of the fungal strains were assessed by methylene blue assay (Oliver et al., 1989). Briefly, the fungal strains were fixed by adding 100 μL of 100% methanol and incubated at room temperature for 30 min. Subsequently, the fixative was aspirated, and 100 μL of filtered 1% (w/v) methylene blue in 0.01 M borate buffer at pH 8.5 was added. After 30 min of incubation at room temperature, the methylene blue buffer was aspirated and rinsed with deionized water. Afterward, the methylene blue stained on the fungal cell wall was eluted in 100 μL of 50 mM hydrochloric acid in 50% ethanol, and the absorbance was measured at 640 nm.

### Data Mining and Bioinformatics Analysis

The database search was performed using GenBank and OneKP (OneKP, 2015). The ginkgotide precursor sequences were obtained by translating the expressed sequence tag (EST) of the *G. biloba* male leaf from GenBank using EMBOSS Transeq (McWilliam et al., 2013). The accession numbers for the ginkgotide precursors are as follows: *gb1* (SRX087421), *gb2* (EX935043.1), *gb3* (CB075727.1), *gb4* (CB094363.1), and *gb5* (DR074391.1). The open reading frame was defined as the region between the specified start (ATG) and stop (TAA, TAG, and TGA) codons. The cleavage site of the signal peptide in the precursor sequence was determined by SignalP 4.0 (Petersen et al., 2011). The isoelectric point was predicted using the ProtParam tool (Wilkins et al., 1999). The alignment of the primary amino acid sequences and precursor sequences was performed using MUSCLE (Edgar, 2004). The sequence logo and phylogenetic tree were generated using iTOL (Letunic and Bork, 2011) and WebLogo (Crooks et al., 2004), respectively.

## Results and Discussion

### Ginkgotide Identification and Purification

**FIGURE 2:**
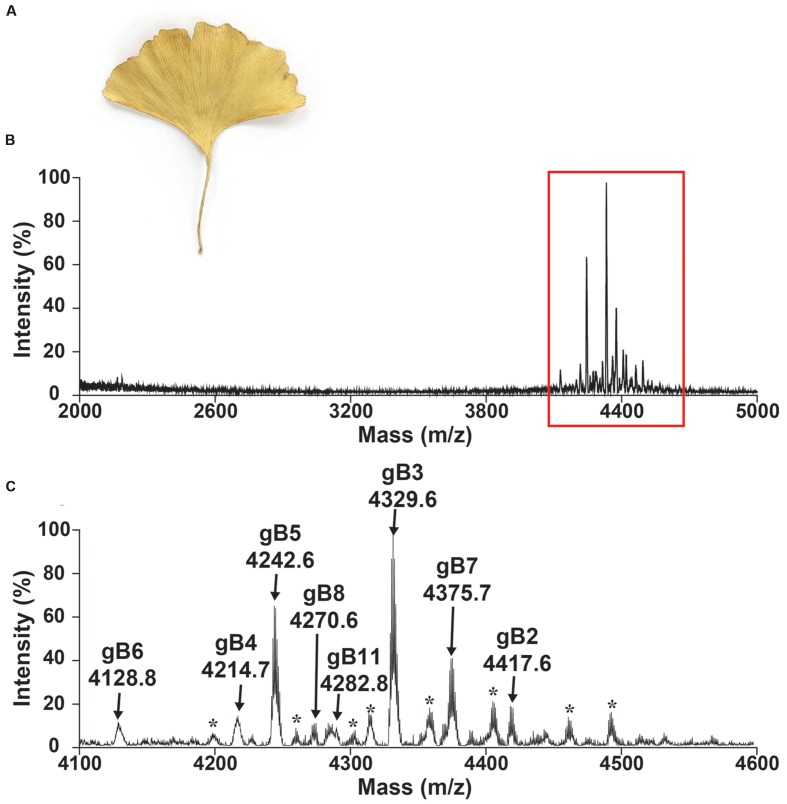
**Mass spectrum of dried *Ginkgo biloba* leaf.** Photograph of **(A)** dried *G. biloba* leaf. MALDI-TOF MS profile of the dried *G. biloba* leaf extract in mass range of **(B)** 2000–5000 and **(C)** 4100–4600 Da. The dried *G. biloba* leaf powder (100 mg) was extracted with 1 mL of water. After centrifuged, the supernatant was fractionated using C18 Ziptip and eluted using 80% ethanol. gB1 were not detected but were later isolated in the preparative HPLC. gB9 and gB10 can only be found in the transcriptomic level. ^∗^ Unknown compounds. Further characterization is required to confirm the identities of these compounds.

A mass-spectrometry-driven approach was used to screen CRPs of medicinal plants in the mass range of 2–6 kDa. To simulate decoction conditions, dried *G. biloba* leaves (**Figure 2**) were extracted with boiling water in a 1:10 ratio (g/mL) and profiled using MALDI-TOF MS in a range between 2 and 5 kDa as illustrated in **Figure 2B**. **Figure 2C** shows an MS profile with strong m/z intensity between 4.1 and 4.6 kDa. Eleven CRPs, designated as ginkgotides gB1 to gB11, were detected. Their identities as CRPs and the number of cysteine residues present were confirmed using a mass shift experiment. The samples were reduced by dithiothreitol and their putative free sulfhydryls were alkylated with iodoacetamide. The mass difference before and after the reductive *S*-alkylation of ginkgotides was monitored using MALDI-TOF MS. Since each *S*-alkylated Cys caused a mass increase of 58 Da, ginkgotides gB3, gB5 and gB8 displayed mass shifts of 464 Da, suggesting the presence of eight Cys residues in each peptide (**Supplementary Figure S1**).

In a scale-up purification using 2 kg of dried *G. biloba* leaves, five high-abundant ginkgotides (gB1, gB2, gB3, gB5, and gB8) were isolated by a series of chromatography steps (see General Experimental Procedures). The relative monoisotopic molecular masses [M+H]^+^ of gB1, gB2, gB3, gB5, and gB8 were 4715.4, 4417.6, 4329.6, 4242.6, and 4270.6 Da, respectively. The yield per kg of dried leaves for these major ginkgotides were approximately 2, 3, 4, 5, and 2 mg, respectively. Gingkotides gB4, gB6, gB7, and gB 9–11 were detected in the chromatography fractions and profiled by MALDI-TOF MS. However, no further purification was conducted due to the relatively low abundance. Ginkgotide gB5 was chosen as a representative for *de novo* sequencing, structural determination, stability and anti-fungal assay because of its high abundance and sequence homology with other ginkgotides.

### Ginkgotide Primary Structure and Conserved Domain

**FIGURE 3:**
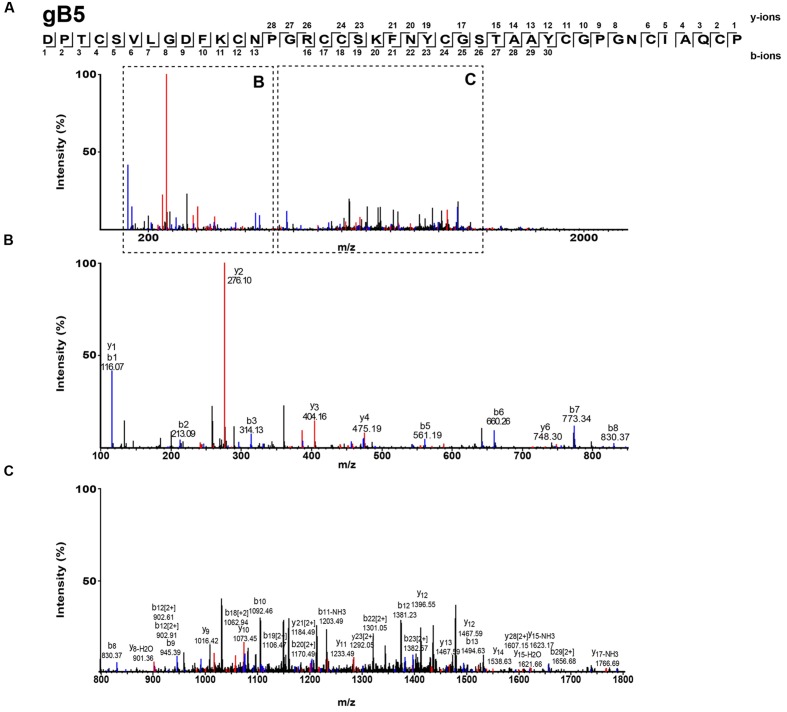
**Mass spectra of ginkgotides gB5 from LC-ESI-LTQ-Orbitrap MS/MS in positive ion mode.** The purified gB5 was *S-*reduced by 20 mM dithiothreitol, *S-*alkylated with 200 mM iodoacetic acid and subsequently de-salted by C18 Ziptip. The spectrum was scanned between mass ranges of **(A)** 100 and 2000 m/z, **(B)** 100 and 900 m/z, and **(C)** 800 and 1800 m/z. Assignment of isobaric amino acids such as Leu/Ile were confirmed by the transcriptome.

The *S*-alkylated ginkgotides were subjected to nanospray MS/MS for *de novo* sequencing. The primary sequence was deduced by evaluating the mass difference between the *b*- and *y*-series ions (**Figure 3**). MS/MS analysis of the *S-*alkylated gB5 revealed a putative sequence of DPTCSV*X*GDFKCNPGRCCS*X*FNYCGSTAAYCGPGNCXA*X*CP, where *X* represents the isobaric residues Ile/Leu or Lys/Gln. Their assignment was resolved by cDNA sequences from GenBank to arrive at the full sequence of gB5 as DPTCSVLGDFKCNPGRCCSKFNYCGSTAAYCGPGNCIAQCP. In addition, there was a general agreement of the deduced (4241.6 Da) and calculated (4241.7 Da) molecular mass. The primary sequences of other ginkgotides were determined similarly (**Supplementary Figures S2** and **S3**).

Analysis of the primary ginkgotide sequences led to two important clues for their classification as a plant CRP family (Tam et al., 2015). The first was the analysis of the ginkgotide cysteine spacing, which forms a specific cysteine network to serve a structural function. Ginkgotides have a highly conserved cysteine pattern of C-C-CC-C-C-C-C with a diagnostic CC motif (adjacent cysteine) at the third and fourth positions. Such cysteine spacing was found in 8C-hevein-like peptides and cystine knot *α*-amylase inhibitors.

The second clue came from the sequence analysis of the non-cysteinyl residues of all 11 ginkgotides for conserved domains. A conserved domain generally serves a specific function associated with a particular CRP family. To determine whether ginkgotides share similar domain architecture with that of other CRPs, a conserved domain search from the NCBI database was performed (Marchler-Bauer et al., 2009). Our search results revealed that a carbohydrate-binding site, namely, hevein or a class 1 chitin-binding domain, was present in all of the ginkgotides studied. This domain is involved in the binding of chitin and has been found in hevein and hevein-like peptides (Tam et al., 2015), plant endochitinases (Collinge et al., 1993) and zymocin, a tRNA endonuclease (Jablonowski and Schaffrath, 2007). Taken together, our search suggested that ginkgotides belong to the subfamily of the 8C-hevein like peptides consisted of 40–45 amino acids.

### Novel Pro-rich 8C-Hevein-Like Peptides

The five members in 8C-hevein-like peptide subfamily include hevein from the latex of rubber trees *Hevea brasiliensis* (Archer, 1960), Fa-AMP1 and Fa-AMP2 from buckwheat *Fagopyrum esculentum* (Fujimura et al., 2003) and Pn-AMP1 and Pn-AMP2 from the Japanese morning glory *Pharbitis nil* (Koo et al., 1998). A comparison of the ginkgotides with other 8C-hevein-like peptides revealed major differences (**Table 1**). The presence of three to six Pro residues in ginkgotides is unusual compared to other 8C-hevein-like peptides, which normally contain zero residues in Pn-AMP1 and Pn-AMP2 or two residues in hevein (loops 2 and 5) and Fa-AMP1 and Fa-AMP2 (loops 2 and 4). Another feature in ginkgotides is the presence of Pro flanking both the N- and C-terminal ends, a sequence motif which has not been previously observed but would have significance in terms of metabolic stability (see later section). However, there is no significant difference in the overall charge and isoelectric point between ginkgotides and those of other 8C-hevein-like peptides.

**Table 1:** Comparison of the primary peptide sequences and physiochemical properties of ginkgotides and 8C-hevein-like peptides.

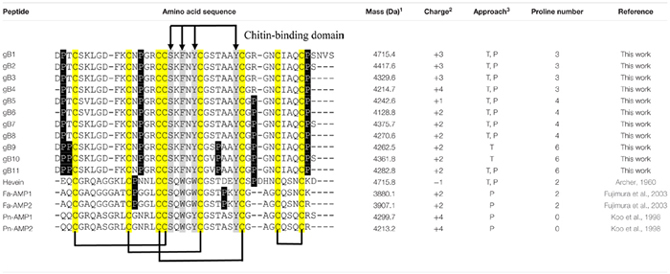

*^1^Mass (Da) = experimental mass. ^2^Charge: the total charge is the sum of positive (lysine, arginine, and histidine residues) and negative (glutamate and aspartate residues) charges present in the sequence. ^3^Approach: the primary sequence was obtained by transcriptomic (T) and/or proteomic (P) approach. The Cys, Pro and chitin-binding domain were highlighted in yellow, black and gray, respectively. Assignment of isobaric amino acids such as Leu/Ile were confirmed by the transcriptome.*

### Conserved Chitin-Binding Domain

**FIGURE 4:**
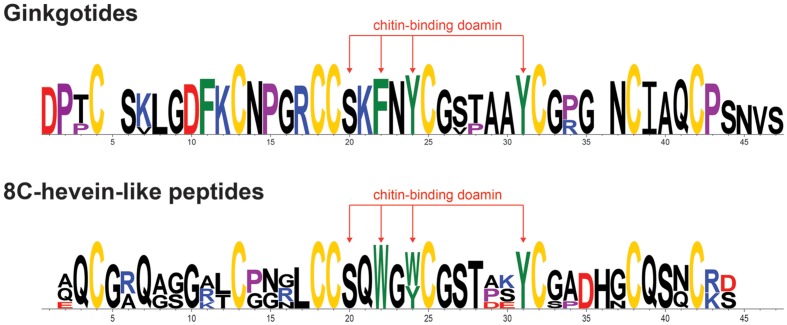
**Sequence analysis of the aligned ginkgotides, hevein and 8C-hevein-like peptides.** The overall height of the stack indicates the sequence conservation, whereas the height of symbols within the stack indicates the relative frequency of each amino at that position. The cysteine, proline, negatively charged (D and E), positively charged (H, K, and R) and aromatic amino acid residues (F, W, and Y) were yellow, violet, red, blue, and green, respectively.

The chitin-binding domain is highly conserved and is comprised of a specific **S*X*ϕ*X*ϕCG*X*_4_Y** (***X*** = small aa and **ϕ** = Y or W) motif located between the intercysteine loops 3 and 4 (**Table 1**). The presence of one serine and three aromatic amino acids within the chitin-binding domain is responsible for the binding affinity toward chitin (Asensio et al., 2000). **Figure 4** shows the sequence logo obtained from the aligned primary sequences of ginkgotides and other 8C-hevein-like peptides. The overall height of the stack indicates the sequence conservation, whereas the symbol heights within the stack indicate the relative frequency of each amino acid at that position (Crooks et al., 2004). Ser and the third aromatic amino acid were conserved among ginkgotides and 8C-hevein-like peptides. In contrast, the first and second aromatic amino acids varied among them, in which Phe and Tyr are found in ginkgotides whereas Trp and Trp/Tyr are found in other 8C-hevein-like peptides, respectively.

### Chitin-Binding Activity of Ginkgotides

To evaluate whether the three-dimensional structure is essential for binding chitin, both the native and *S*-alkylated ginkgotides were used in the chitin-binding assay (**Supplementary Figure S4**). After a 4-h incubation with chitin beads, the ginkgotides (gB3, gB5, and gB8) were absent in the supernatant but were recovered in the acidic elution buffer. These results suggest that the ginkgotides have a chitin-binding affinity. In contrast, the *S*-alkylated ginkgotides were detected in the supernatant instead of the acidic elution buffer, suggesting that the tertiary structure was essential to their chitin-binding ability.

### Ginkgotide NMR Structure

**FIGURE 5:**
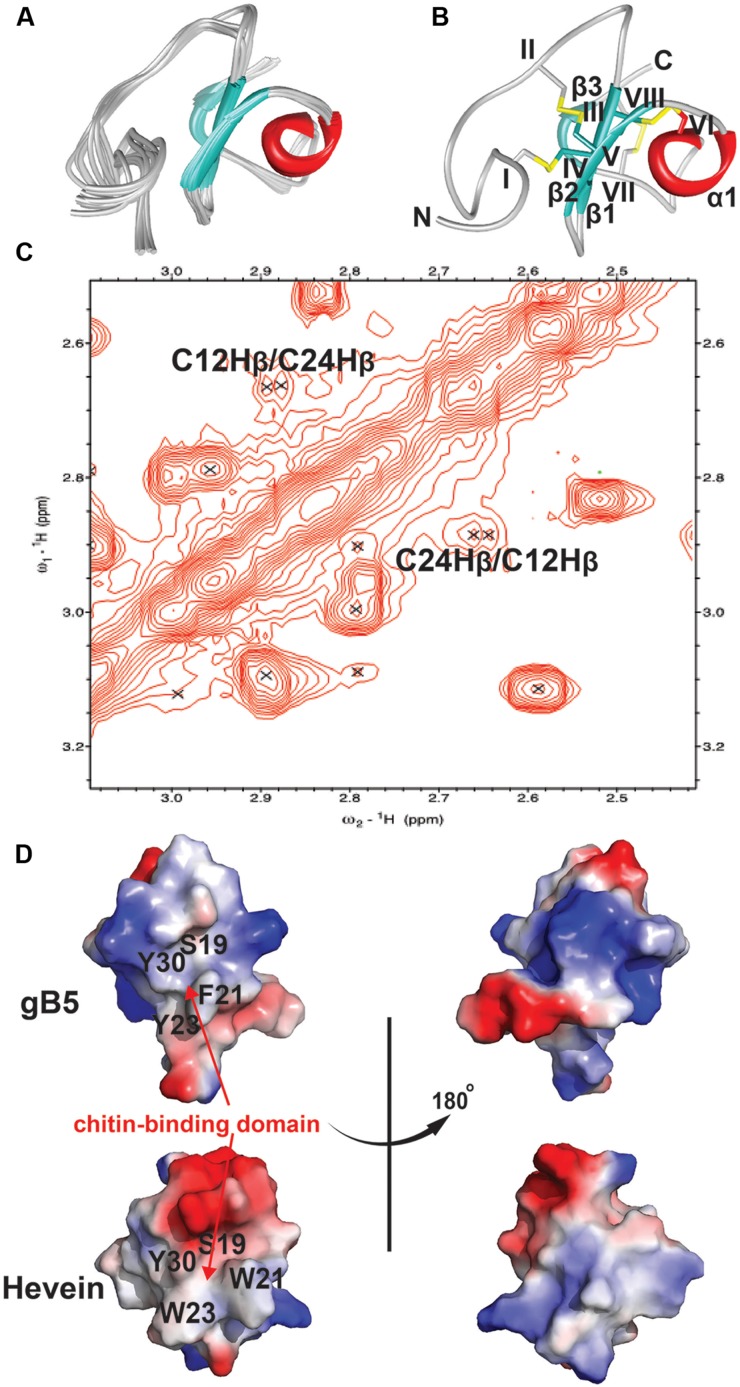
**2D NOESY spectrum and 3D structure of gB5.** **(A)** Superposition of the gB5 backbone traces from the final 20 ensembles solution structures and restrained energy minimized structure. **(B)** The ribbon representation of the gB5 structure. The disulfide bonds are formed between CysI-CysIV, CysII-CysV, CysIII-CysVI, and CysVII-CysVIII. **(C)** The NOE cross peaks between Hβs of Cys12 and Cys24, displayed by Sparky 3.115. **(D)** Electrostatic surface of gB5 and hevein (PDB: 1HEV) in two views revealing the chitin-binding domain. The distribution of electrostatic charges was illustrated in red (negatively charged), blue (positively charged) and white (neutral). The residues (gB5: S19, F21, Y23, and Y30; hevein: S19, W21, W23, and Y30) have been shown to play an important role in binding toward chitin.

The NMR solution structure of gB5, as shown in **Figure 5**, was determined using the distance restraints obtained from 2D ^1^H-^1^H-TOCSY and NOESY, as well as the hydrogen bond restraints based on the H/D exchange NMR experiment. All spin-spin systems of gB5 were identified, and approximately 98% of the proton resonances were unambiguously assigned. The solution structure of gB5 was determined based on a total of 439 NMR-derived distance restraints and six hydrogen bonds. **Figure 5A** shows the NMR ensemble of the 20 lowest-energy gB5 structures. The root-mean-square deviation (RMSD) value of the 20 best structures for residues Asp9–Pro41 was 0.49 ± 0.17 Å, and that for all heavy atoms was 0.99 ± 0.20 Å (**Supplementary Table S1**). The structure of gB5 was well-defined by a number of medium- and long-range NOEs, which consisted of three short extended anti-parallel β-strands (β1: Cys17–Ser19; β1: Cys24–Gly25; and β1: Cys37–Ala39) and a one-turn α-helix (α1: Ala28–Cys31) (**Figure 5B**). The N-terminus of gB5 has no secondary structure (Asp1–Arg16). The four Pro at positions 2, 14, 33, and 41 were all identified in *trans*-conformation based on the presence of H^δ^_(Proi)_ – H^α^_(i-1)_ NOE cross-peaks (**Supplementary Figure S5**). The two negatively charged residues (Asp1 and Asp9) of gB5 were clustered at the N-terminus, whereas the three positively charged residues (Lys11, Arg16, and Lys20) were distributed around the strand β*1*. The three anti-parallel β-strands and the α-helix consisted entirely of hydrophobic residues. PROCHECK analysis suggested that all residues were distributed in the allowed region of the Ramachandran map (**Supplementary Table S1**).

To confirm the disulfide connectivity of gB5, the structural energies of 15 different disulfide bond patterns were calculated by CNSsolve 1.3. Among the eight Cys, there were *d_ββ_*(*i. j*) and *d_αβ_*(*i. j*) NOEs between the two closest in space. For Cys12 and Cys24 (CysII–CysV), the NOE cross peak between H*_β_s* was unambiguous (**Figure 5C**). For the rest, there was ambiguity due to the chemical shifts that were similar to those of other protons. In this case, the different combinations between the remaining six Cys were simulated in a structure calculation in which the disulfide bond between Cys12 and Cys24 was fixed. The structural energy between different disulfide combinations revealed that pattern 1 (CysI–CysIV, CysII–CysV, CysIII–CysVI, and CysVII–CysVIII) had the lowest energy (546.74 ± 9.17 kcal/mol) among other disulfide bond patterns with an energy ranging between 566.73 and 1442.84 kcal/mol (**Supplementary Tables S2** and **S3**), suggesting that it is more energetically favorable than the other possible combinations. This conformation was in agreement with the disulfide connectivity of hevein (PDB: 1HEV), as determined by NMR (Andersen et al., 1993) and X-ray crystallography (Reyes-Lopez et al., 2004), which included a cystine knot motif in the N-terminus with the fourth disulfide bond located at the C-terminus. The disulfide bonds CysI–CysIV and CysII–CysV link the N terminal loop and the β*1* and β*2* strands, respectively. The disulfide bond CysIII–CysVI makes the α-helix close to the strand β*1*. The disulfide bond CysVII–CysVIII links the N- and C-termini of the last strand β*3*. As shown in **Figure 5D**, the topography of surface electrostatic charge suggested that the chitin-binding residues (Ser19, Phe21, Tyr23, and Tyr30) in gB5 are relatively neutral, and most of the charged residues (highlighted in red and blue for the negatively and positively charged amino acids, respectively) were located on the opposite side of the chitin-binding domains. It is speculated that the distribution of the charged residues might alter the ability of hevein-like peptides to bind chitin; however, further experiments to verify this issue are urgently warranted.

### Thermal, Acidic, and Enzymatic Stability of Ginkgotides

**FIGURE 6:**
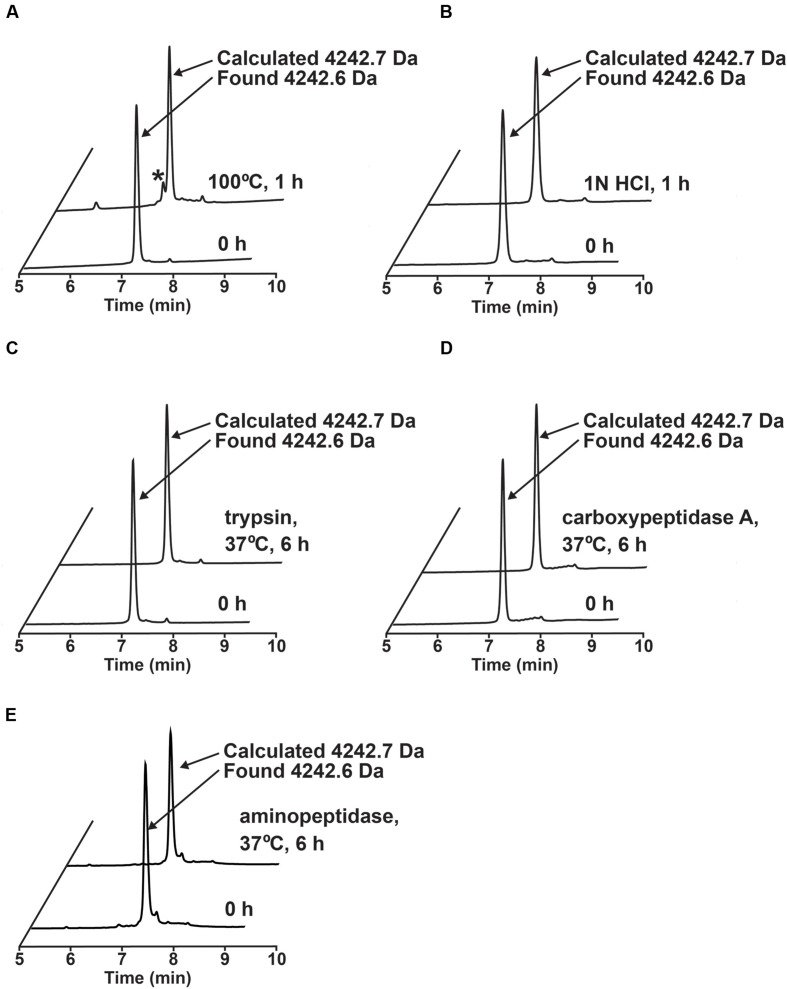
**Stability assays of ginkgotide gB5.** **(A)** Thermal stability of gB5 incubated at 100°C for 1 h. ^∗^ Represents the degraded products. **(B)** Acidic condition stability of gB5 incubated in 1N HCl (pH 2.0) for 1 h. Exo- and endo-peptidase enzymatic stability of gB5 against **(C)** trypsin, **(D)** carboxypeptidase and **(E)** aminopeptidase, respectively for 6 h in buffer as suggest by manufactures at 37°C. The molecular weight of the peak was determined by MALDI-MS.

The stability of ginkgotide gB5 against heat, acid, endopeptidase and exopeptidases was determined by RP-HPLC. As shown **Figure 6**, gB5 displayed a high tolerance to boiling water (100°C) and acidic conditions (1 M HCl at pH 2.0) for 1 h, with 80 and 92.3% of the peptide remaining, respectively. Furthermore, gB5 was resistant to digestion with the endopeptidase trypsin and exopeptidase carboxypeptidase A and aminopeptidase for up to 6 h with >95% of the peptide remaining. This stability is because of the presence of highly cross-linked structure by disulfide bonds, which prevents hydrolysis of peptide bond under harsh conditions and hinders the formation of enzyme-substrate complex. The resistance to trypsin digestion is in agreement with other plant cystine knot CRPs that contain a compact structure (Nguyen et al., 2011a,b, 2012, 2013, 2014, 2015; Kini et al., 2015). The pair of Pro residues flanking both the N- and C-termini could account for its extraordinary resistance to proteolysis by carboxypeptidase A and aminopeptidase. The ability of ginkgotides to withstand harsh treatments suggests that they could be relevant as active compounds in traditional medicines, which are served as decoctions and are taken orally.

### Ginkgotide Anti-fungal Activities

The effect of ginkgotide gB5 against fungal mycelium growth was screened using a disk diffusion assay. Crescent-shaped zones were observed around the gB5-treated disk, suggesting an anti-fungal activity against *Aspergillus niger. C. lunata. Fusarium oxysporum*, and *R. solani* (**Supplementary Figure S6**). To further evaluate the potency of its anti-fungal properties, the half maximal inhibitory concentration (IC_50_) was determined using a micro-broth dilution assay. After 24-h treatments at 25°C, the IC_50_ values of gB5 against *A. niger. C. lunata. F. oxysporum*, and *R. solani* were 6.8, 10.0, 69.2, and 20.0 μg/mL, respectively. Our results are in agreement with previous reports on the anti-fungal activity of Pn-AMP1, Fa-AMP1, and EAFP1 against various fungal strains, with IC_50_ ranges of 5–26, 11–36, and 35–155 μg/mL, respectively (Koo et al., 1998; Van den Bergh et al., 2002a; Fujimura et al., 2003).

**FIGURE 7:**
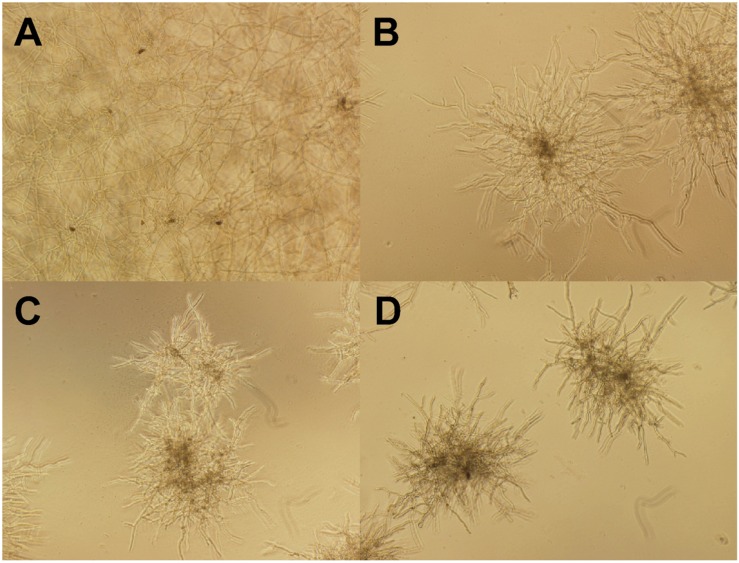
**Microscopic views of the mycelium growth of *Curvularia lunata* (100X).** The well without the addition of gB5 **(A)** was used as a control and were co-incubated with **(B)** 12.5, **(C)** 25 and **(D)** 50 μg/mL gB5 in half strength potato dextrose broth at 25°C for 24 h.

**Figure 7** shows the morphological changes of *C. lunata* before and after gB5 treatment (12.5–50 μg/mL) cultured in half-strength potato dextrose broth at 25°C for 24 h. Compared with the control experiment (**Figure 7A**), the addition of gB5 resulted in shorter and highly branched hypahae with swollen hyphal tips as well as germination of the fungal spores in a concentration-dependent manner (**Figures 7B–D**). These fungal morphological changes have also been observed in the anti-fungal experiments treated with 6C- and 10C-hevein-like peptides IWF4 from *Beta vulgaris* (Nielsen et al., 1997) and Ee-CPB from *E. europaeus*, respectively (Van den Bergh et al., 2002a,b).

Two mechanisms have been suggested for the anti-fungal activity exerted by hevein-like peptides. First, the presence of a chitin-binding domain allows these peptides to bind to chitinous fungal cell walls. This prevents cross-linking of nascent chitin chains and β-glucan microfibrils, resulting in a disturbance of cell wall morphogenesis and eventually inhibition of hyphal growth (Nielsen et al., 1997). Second, the small footprint and compact structure of the hevein-like peptides allows them to migrate through the pores of the fungal cell wall and interact with the fungal plasma membrane. Hevein-like peptides may interact with surface glyco-conjugates or alternate membrane polarity, leading to leakage of cytoplasmic materials and subsequently causing breakage of the fungal cell wall (Van den Bergh et al., 2002a,b). This membrane leakage mechanism has been suggested for the antifungal activity of other CRP families such as thionins (Florack and Stiekema, 1994) and defensins (Thevissen et al., 1996).

### Ginkgotide Biosynthesis

**FIGURE 8:**
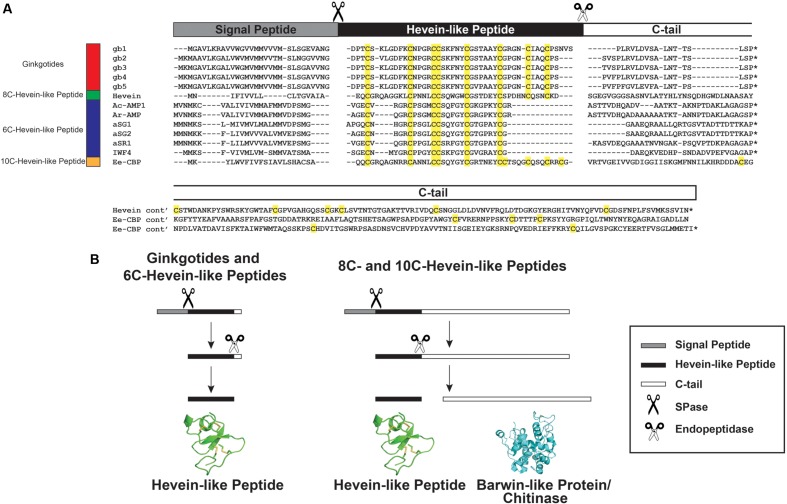
**Gene alignment and biosynthesis pathway of hevein-like peptides.** **(A)** Precursor sequences alignment of ginkgotides, hevein, and hevein-like peptides. The precursors are divided into three major domains, including the signal peptide, mature hevein-like peptide domain and C-terminal tail. **(B)** Comparison of the biosynthesis pathways between ginkgotides and other hevein-like peptides. Signal peptide was removed from the full precursor sequences by SPase. The C-terminal tail is then cleaved by endopeptidase to release the mature peptides. In 8C- (except ginkgotides) and 10C-hevein-like peptides, the C-terminal tail was coded for a bio-functional protein such as barwin-like protein or chitinase. ^∗^ Represents the stop codon.

A GenBank transcriptomic search revealed five full-length ginkgotide precursor sequences including *gb1. gb2. gb3. gb4* and *gb5*, which encode for ginkgotides gB1–4, gB 5–6, gB 7–8, gB 9, and gB10–11, respectively. **Figure 8** shows their alignment with precursor sequences for hevein (Broekaert et al., 1990) and hevein-like peptides including Ac-AMP1 from *Amaranthus caudatus* (De Bolle et al., 1996), Ar-AMP1 from *Amaranthus retroflexus* (Lipkin et al., 2005), aSG1 and aSG2 from *Alternanthera sessilis* var. green and aSR1 from *Alternanthera sessilis* var. red (Kini et al., 2015) IWF4 from *B. vulgaris* (Nielsen et al., 1997), and Ee-CBP from *E. europaeus* (Van den Bergh et al., 2004). These precursors shared the same three-domain architecture, which includes an endoplasmic reticulum (ER) signal peptide, a mature hevein-like peptide domain and a C-terminal tail. This precursor arrangement suggests that ginkgotides are secretory peptides similar to other hevein-like peptides subfamilies. Secretory peptides are exported from the cytoplasm with a signal peptide, which is responsible for routing the peptides to the ER membrane. After the precursor is translocated, the signal peptide is cleaved by signal peptidase (SPase). The signal peptide cleavage site in ginkgotides was highly conserved. The peptide bond between Gly and Asp was cleaved, and the bonds in other hevein-like peptides were between Gly/Ala and small and polar residues such as Ala/Val and Glu/Gln, respectively (**Figure 8A**). Subsequently, the C-terminal tail was cleaved by endopeptidase in the ER, and the mature peptide was released for further post-translational modifications.

As shown in **Figure 8B**, a distinguishing feature of ginkgotides is the short C-tail (20 aa) compared with those of other 8C- and 10C-hevein-like peptides, which carry a functional protein cargo. In hevein, the C-tail encoded for a Barwin-like protein (126 aa; an N-acetylglucosamine-binding protein) (Broekaert et al., 1990), whereas in Ee-CBP, it encoded for a class I chitinase (254 aa; a glycosyl hydrolase) (Van den Bergh et al., 2002b, 2004). These functional protein cargos have been shown to possess antifungal activities similar to those of hevein and hevein-like peptides (Broekaert et al., 1990; Van den Bergh et al., 2004; Lipkin et al., 2005).

A recent study revealed that co-treatment with Ee-CBP and Ee-chitinase (a class 1 chitinase from *E. europaeus*) displays a significant synergistic antifungal effect (Van den Bergh et al., 2004). The synergism is due to the combination of different modes of actions, where Ee-chitinase degrades the nascent chitin and makes the chitin microfibrils more accessible to Ee-CBP, resulting in a disturbance of cell wall morphogenesis and hyphal growth (Van den Bergh et al., 2002b, 2004). We speculated that the co-expression of hevein-like peptides and functional proteins from the same mRNA will possess a similar synergistic effect. Thus far, this chimeric arrangement has been found in only 8C- and 10C-hevein-like peptides and was absent in ginkgotides and 6C-hevein-like peptides. Since *G. biloba* is a less evolved taxon compared to angiosperms, we speculated that the prototypic C-terminal tail in hevein-like peptides is protein-cargo-free, where the addition of a functional protein is due to convergent evolution. In contrast, mRNA deletion resulted in the absence of the fourth disulfide bond and functional protein in 6C-hevein-like peptides, which have been observed in only the Amaranthaceae family.

### Data Mining and Phylogenetic Analysis of Pro-rich 8C-Hevein-Like Peptides *In Planta*

To determine the distribution of Pro-rich hevein-like peptides *in planta*, a tBLASTn search using ginkgotides as a query was performed. The result revealed 85 sequence homologs from 47 different plants with an E-value < 0.005. However, not all of the homologs contained the unique features of the Pro-rich 8C-hevein-like peptide. A manual filtering was performed to remove sequences with the following criteria: odd number of Cys residues (1); signal peptides with less than 10 residues (3); undetermined/ untranslated amino acids (3); identical sequence from the same plant (9); and lack of Pro flanks at N- and C-terminal ends (17), where the breket represents the number of sequences. Subsequently, 52 precursor sequences were identified, which encoded for 42 putative Pro-rich 8C-hevein-like peptides (**Supplementary Table S4**). Six of the putative peptides were expressed in multiple plants, which had identical mature domains but different signal peptides and/or C-tails. For example, cD1 was expressed in *Calocedrus decurrens. Cunninghamia lanceolata. Cupressus dupreziana. Platycladus orientalis. Sequoiadendron giganteum*, and *Taiwania cryptomerioides*.

**FIGURE 9:**
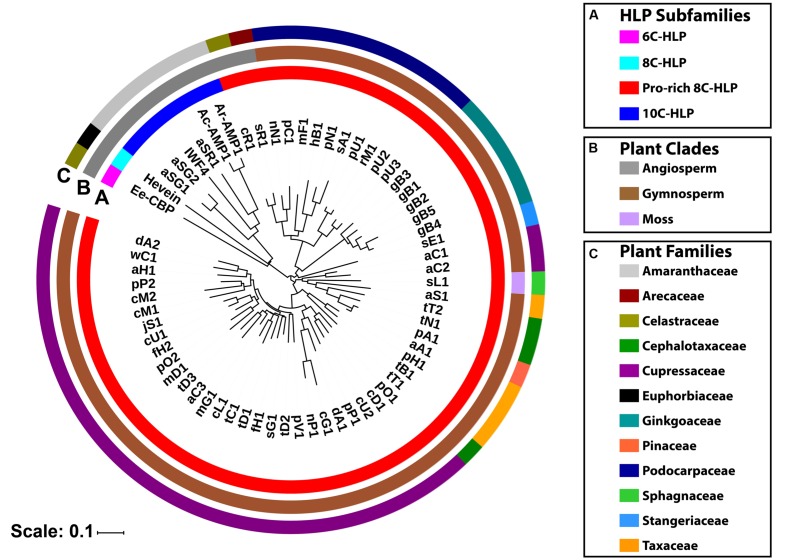
**Phylogenetic tree of hevein-like peptides.** The precursor sequences were aligned by MUSCLE and the phylogenetic tree was generated by iTOL. Classification based on **(A)** the number of cysteine residues in the mature domain, **(B)** plant’s clade, and **(C)** plant’s family.

These ginkgotide homologs contain 43–50 amino acids, of which 3–5 residues are Pro. The aligned precursor sequences were analyzed by neighbor-joining clustering algorithm and displayed as a phylogenetic tree in **Figure 9**. Two major cluster were obtained, in which Pro-rich 8C-hevein-like peptides (highlighted in red) were separated from other hevein-like peptides (**Figure 9A**). Within the angiosperm cluster, the 8C- and 10C-hevein-like peptides form separate clusters that were separated from the 6C-hevein-like peptides. As shown in **Figures 9B,C**, three homologs, including sL1 from the byrophyta (moss) and sR1 and cR1 from the angiosperm, were classified into the gymnosperm cluster due to their high abundance of Pro residues and high sequence similarity to the other gymnosperm members. Of the 52 ginkgotide homolog precursor sequences, 49 are derived from gymnosperms and distributed in six families, including Cephalotaxacea, Cupressaceae, Pinaceae, Podocarpaceae, Stangeriaceae, and Taxaceae. Together, data mining of the transcriptomic data and phylogenetic analysis revealed that ginkgotides and their homologs belong to a new class of 8C-hevein-like peptides, are distributed mainly in gymnosperms and occasionally in mosses and angiosperms.

## Conclusion

Here, we report the discovery and characterization of a new class of hevein-like peptides, ginkgotides, from *G. biloba* leaves. Ginkgotides exhibit the following characteristics: (1) 40–45 residues and three to six Pro; (2) Pro residues flanking both the N- and C-terminal ends; (3) a highly conserved chitin-binding domain located between intercysteine loops 3 and 4; (4) membership in the subfamily of 8C-hevein-like peptides with a cystine knot and an additional disulfide bond at the C-terminus; (5) potential to serve as a scaffold for peptide engineering because of their high tolerance for thermal, acidic, exo- and endo-peptidase degradation; (6) a significantly short and non-chimeric C-terminal tail and (7) typically found in gymnosperms. Taken together, ginkgotides belong to a new class of the 8C-hevein-like peptides subfamily.

Data mining revealed an additional 42 putative Pro-rich 8C-hevein-like peptides *in planta*, and our findings expanded the existing 8C-hevein-like peptide library from five to 58. Our discovery of Pro-rich 8C-hevein-like peptides enriches the existing library of hevein-like peptides and provides insights into their structure, biosynthesis, occurrence, and distribution *in planta*.

## Author Contributions

JT, KW, and WT conceived and designed the experiments. KW, WT, AS, and TX performed the experiments, analyzed the data, and wrote the manuscript. JT, SS, and DY revised the manuscript. All authors read and approved the final version of the manuscript.

## Conflict of Interest Statement

The authors declare that the research was conducted in the absence of any commercial or financial relationships that could be construed as a potential conflict of interest.

## Footnotes

1 http://www.ebi.ac.uk/thornton-srv/databases/pdbsum/Generate.html

## References

[B1] Al-Achi A. (2008). *An Introduction to Botanical Medicines: History, Science, Uses, and Dangers*. Westport, CT: Praeger Publishers.

[B2] Andersen N. H. Cao B. Rodriguez-Romero A. Arreguin B. (1993). Hevein: NMR assignment and assessment of solution-state folding for the agglutinin-toxin motif. *Biochemistry* 32 1407–1422. 10.1021/bi00057a004 8431421

[B3] Archer B. L. (1960). The proteins of *Hevea brasiliensis* latex. 4. Isolation and characterization of crystalline hevein. *Biochem. J.* 75 236–240. 10.1042/bj0750236 13794068 PMC1204415

[B4] Asensio J. L. Canada F. J. Siebert H. C. Laynez J. Poveda A. Nieto P. M. (2000). Structural basis for chitin recognition by defense proteins: GlcNAc residues are bound in a multivalent fashion by extended binding sites in hevein domains. *Chem. Biol.* 7 529–543. 10.1016/S1074-5521(00)00136-8 10903932

[B5] Bent S. (2008). Herbal medicine in the United States: review of efficacy, safety, and regulation: grand rounds at University of California, San Francisco Medical Center. *J. Gen. Intern. Med.* 23 854–859. 10.1007/s11606-008-0632-y 18415652 PMC2517879

[B6] Broekaert I. Lee H. I. Kush A. Chua N. H. Raikhel N. (1990). Wound-induced accumulation of mRNA containing a hevein sequence in laticifers of rubber tree (*Hevea brasiliensis*). *Proc. Natl. Acad. Sci. U.S.A.* 87 7633–7637. 10.1073/pnas.87.19.7633 2217194 PMC54802

[B7] Broekaert W. F. Cammue B. P. A. Debolle M. F. C. Thevissen K. Desamblanx G. W. Osborn R. W. (1997). Antimicrobial peptides from plants. *Crit. Rev. Plant Sci.* 16 297–323. 10.1080/713608148

[B8] Brunger A. T. Adams P. D. Clore G. M. Delano W. L. Gros P. Grosse-Kunstleve R. W. (1998). Crystallography & NMR system: a new software suite for macromolecular structure determination. *Acta Crystallogr. D Biol. Crystallogr.* 54 905–921. 10.1107/S0907444998003254 9757107

[B9] Cammue B. P. De Bolle M. F. Schoofs H. M. Terras F. R. Thevissen K. Osborn R. W. (1994). Gene-encoded antimicrobial peptides from plants. *Ciba Found. Symp.* 186 91–101. 10.1002/9780470514658.ch6 discussion 101–106 7768160

[B10] Chaw S. M. Zharkikh A. Sung H. M. Lau T. C. Li W. H. (1997). Molecular phylogeny of extant gymnosperms and seed plant evolution: analysis of nuclear 18S rRNA sequences. *Mol. Biol. Evol.* 14 56–68. 10.1093/oxfordjournals.molbev.a025702 9000754

[B11] China Pharmacopoeia Commission (2010). *Pharmacopoeia of the People’s Republic of China*. Beijing: China Medical Science Press.

[B12] Collinge D. B. Kragh K. M. Mikkelsen J. D. Nielsen K. K. Rasmussen U. Vad K. (1993). Plant chitinases. *Plant J.* 3 31–40. 10.1007/978-3-0348-9225-4_5 8401605

[B13] Crooks G. E. Hon G. Chandonia J. M. Brenner S. E. (2004). WebLogo: a sequence logo generator. *Genome Res.* 14 1188–1190. 10.1101/gr.849004 15173120 PMC419797

[B14] De Bolle M. F. Osborn R. W. Goderis I. J. Noe L. Acland D. Hart C. A. (1996). Antimicrobial peptides from *Mirabilis jalapa* and *Amaranthus caudatus*: expression, processing, localization and biological activity in transgenic tobacco. *Plant Mol. Biol.* 31 993–1008. 10.1007/BF00040718 8843942

[B15] Delaglio F. Grzesiek S. Vuister G. W. Zhu G. Pfeifer J. Bax A. (1995). NMRPipe: a multidimensional spectral processing system based on UNIX pipes. *J. Biomol. NMR* 6 277–293. 10.1007/BF00197809 8520220

[B16] Edgar R. C. (2004). MUSCLE: multiple sequence alignment with high accuracy and high throughput. *Nucleic Acids Res.* 32 1792–1797. 10.1093/nar/gkh340 15034147 PMC390337

[B17] Florack D. E. Stiekema W. J. (1994). Thionins: properties, possible biological roles and mechanisms of action. *Plant Mol. Biol.* 26 25–37. 10.1007/BF00039517 7948874

[B18] Fujimura M. Minami Y. Watanabe K. Tadera K. (2003). Purification, characterization, and sequencing of a novel type of antimicrobial peptides, Fa-AMP1 and Fa-AMP2, from seeds of buckwheat (*Fagopyrum esculentum* Moench.). *Biosci. Biotechnol. Biochem.* 67 1636–1642. 10.1271/bbb.67.1636 12951494

[B19] Hammami R. Ben Hamida J. Vergoten G. Fliss I. (2009). PhytAMP: a database dedicated to antimicrobial plant peptides. *Nucleic Acids Res.* 37 D963–D968. 10.1093/nar/gkn655 18836196 PMC2686510

[B20] Hegedus N. Marx F. (2013). Antifungal proteins: more than antimicrobials? *Fungal Biol. Rev.* 26 132–145. 10.1016/j.fbr.2012.07.002 23412850 PMC3569713

[B21] Hong Kong Chinese Materia Medica Standards (2010). *Folium Ginkgo.* Hong Kong: Department of Health, Hong Kong Special Administrative Region.

[B22] Hori T. Ridge R. W. Tulecke W. Tredici P. D. Tremouillaux-Guiller J. Tobe H. (2012). *Ginkgo Biloba A Global Treasure: From Biology to Medicine.* Tokyo: Springer.

[B23] Huang C. C. Couch G. S. Pettersen E. F. Ferrin T. E. (1996). Chimera: an extensible molecular modeling application constructed using standard components. *Pac. Symp. Biocomput.* 1 724.

[B24] Jablonowski D. Schaffrath R. (2007). Zymocin, a composite chitinase and tRNase killer toxin from yeast. *Biochem. Soc. Trans.* 35 1533–1537. 10.1042/BST0351533 18031261

[B25] Kini S. G. Nguyen P. Q. T. Weissbach S. Mallagaray A. Shin J. Yoon H. S. (2015). Studies on the chitin-binding property of novel cysteine-rich peptides from *Alternanthera sessilis*. *Biochemistry* 3 6639–6649. 10.1021/acs.biochem.5b00872 26467613

[B26] Koo J. C. Lee S. Y. Chun H. J. Cheong Y. H. Choi J. S. Kawabata S. (1998). Two hevein homologs isolated from the seed of Pharbitis nil L. exhibit potent antifungal activity. *Biochim. Biophys. Acta* 1382 80–90. 10.1016/S0167-4838(97)00148-9 9507071

[B27] Letunic I. Bork P. (2011). Interactive Tree Of Life v2: online annotation and display of phylogenetic trees made easy. *Nucleic Acids Res.* 39 W475–W478. 10.1093/nar/gkr201 21470960 PMC3125724

[B28] Lipkin A. Anisimova V. Nikonorova A. Babakov A. Krause E. Bienert M. (2005). An antimicrobial peptide Ar-AMP from amaranth (*Amaranthus retroflexus* L.) seeds. *Phytochemistry* 66 2426–2431. 10.1016/j.phytochem.2005.07.015 16126239

[B29] Marchler-Bauer A. Anderson J. B. Chitsaz F. Derbyshire M. K. Deweese-Scott C. Fong J. H. (2009). CDD: specific functional annotation with the conserved domain database. *Nucleic Acids Res.* 37 D205–D210. 10.1093/nar/gkn845 18984618 PMC2686570

[B30] McKenna D. J. Jones K. Hughes K. (2001). Efficacy, safety, and use of ginkgo biloba in clinical and preclinical applications. *Altern. Ther. Health Med.* 7 88–90. 11565403

[B31] McWilliam H. Li W. Uludag M. Squizzato S. Park Y. M. Buso N. (2013). Analysis tool web services from the EMBL-EBI. *Nucleic Acids Res.* 41 W597–W600. 10.1093/nar/gkt376 23671338 PMC3692137

[B32] Nguyen G. K. Lian Y. Pang E. W. Nguyen P. Q. Tran T. D. Tam J. P. (2013). Discovery of linear cyclotides in monocot plant *Panicum laxum* of Poaceae family provides new insights into evolution and distribution of cyclotides in plants. *J. Biol. Chem.* 288 3370–3380. 10.1074/jbc.M112.415356 23195955 PMC3561556

[B33] Nguyen G. K. Lim W. H. Nguyen P. Q. Tam J. P. (2012). Novel cyclotides and uncyclotides with highly shortened precursors from *Chassalia chartacea* and effects of methionine oxidation on bioactivities. *J. Biol. Chem.* 287 17598–17607. 10.1074/jbc.M111.338970 22467870 PMC3366795

[B34] Nguyen G. K. Zhang S. Nguyen N. T. Nguyen P. Q. Chiu M. S. Hardjojo A. (2011a). Discovery and characterization of novel cyclotides originated from chimeric precursors consisting of albumin-1 chain a and cyclotide domains in the Fabaceae family. *J. Biol. Chem.* 286 24275–24287. 10.1074/jbc.M111.229922 21596752 PMC3129208

[B35] Nguyen G. K. Zhang S. Wang W. Wong C. T. Nguyen N. T. Tam J. P. (2011b). Discovery of a linear cyclotide from the bracelet subfamily and its disulfide mapping by top-down mass spectrometry. *J. Biol. Chem.* 286 44833–44844. 10.1074/jbc.M111.290296 21979955 PMC3247958

[B36] Nguyen P. Q. Luu T. T. Bai Y. Nguyen G. K. Pervushin K. Tam J. P. (2015). Allotides: proline-rich cystine knot alpha-amylase inhibitors from *Allamanda cathartica*. *J. Nat. Prod.* 78 695–704. 10.1021/np500866c 25832441

[B37] Nguyen P. Q. Wang S. Kumar A. Yap L. J. Luu T. T. Lescar J. (2014). Discovery and characterization of pseudocyclic cystine-knot alpha-amylase inhibitors with high resistance to heat and proteolytic degradation. *FEBS J.* 281 4351–4366. 10.1111/febs.12939 25040200

[B38] Nielsen K. K. Nielsen J. E. Madrid S. M. Mikkelsen J. D. (1997). Characterization of a new antifungal chitin-binding peptide from sugar beet leaves. *Plant Physiol.* 113 83–91. 10.1104/pp.113.1.83 9008390 PMC158118

[B39] Odintsova T. I. Vassilevski A. A. Slavokhotova A. A. Musolyamov A. K. Finkina E. I. Khadeeva N. V. (2009). A novel antifungal hevein-type peptide from *Triticum kiharae* seeds with a unique 10-cysteine motif. *FEBS J.* 276 4266–4275. 10.1111/j.1742-4658.2009.07135.x 19583772

[B40] Oliver M. H. Harrison N. K. Bishop J. E. Cole P. J. Laurent G. J. (1989). A rapid and convenient assay for counting cells cultured in microwell plates: application for assessment of growth factors. *J. Cell Sci.* 92(Pt 3), 513–518. 2592453 10.1242/jcs.92.3.513

[B41] OneKP (2015). *The One Thousand Plants Project.* Available at: https://sites.google.com/a/ualberta.ca/onekp/home [accessed Oct 10, 2016].

[B42] Petersen T. N. Brunak S. Von Heijne G. Nielsen H. (2011). SignalP 4.0: discriminating signal peptides from transmembrane regions. *Nat. Methods* 8 785–786. 10.1038/nmeth.1701 21959131

[B43] Ponce de Leon I. Montesano M. (2013). Activation of defense mechanisms against pathogens in mosses and flowering plants. *Int. J. Mol. Sci.* 14 3178–3200. 10.3390/ijms14023178 23380962 PMC3588038

[B44] Reyes-Lopez C. A. Hernandez-Santoyo A. Pedraza-Escalona M. Mendoza G. Hernandez-Arana A. Rodriguez-Romero A. (2004). Insights into a conformational epitope of Hev b 6.*02* (hevein). *Biochem. Biophys. Res. Commun.* 314 123–130. 10.1016/J.Bbrc.2003.12.068 14715255

[B45] Rinaudo M. (2006). Chitin and chitosan: properties and applications. *Prog. Polym. Sci.* 31 603–632. 10.1016/j.progpolymsci.2006.06.001

[B46] Schröginger L. L. C. (2015). *The PyMOL Molecular Graphics System, Version 1.8.* New York, NY: Schrödinger.

[B47] Sels J. Mathys J. De Coninck B. M. Cammue B. P. De Bolle M. F. (2008). Plant pathogenesis-related (PR) proteins: a focus on PR peptides. *Plant Physiol. Biochem.* 46 941–950. 10.1016/j.plaphy.2008.06.011 18674922

[B48] Takakura Y. Ito T. Saito H. Inoue T. Komari T. Kuwata S. (2000). Flower-predominant expression of a gene encoding a novel class I chitinase in rice (*Oryza sativa* L.). *Plant Mol. Biol.* 42 883–897. 10.1023/A:1006401816145 10890535

[B49] Tam J. P. Wang S. Wong K. H. Tan W. L. (2015). Antimicrobial peptides from plants. *Pharmaceuticals* 8 711–757. 10.3390/ph8040711 26580629 PMC4695807

[B50] Thevissen K. Ghazi A. De Samblanx G. W. Brownlee C. Osborn R. W. Broekaert W. F. (1996). Fungal membrane responses induced by plant defensins and thionins. *J. Biol. Chem.* 271 15018–15025. 10.1074/jbc.271.25.15018 8663029

[B51] Van Damme E. J. Broekaert W. F. Peumans W. J. (1988). The *Urtica dioica* agglutinin is a complex mixture of isolectins. *Plant Physiol.* 86 598–601. 10.1104/pp.86.2.598 16665952 PMC1054529

[B52] Van den Bergh K. P. Proost P. Van Damme J. Coosemans J. Van Damme E. J. Peumans W. J. (2002a). Five disulfide bridges stabilize a hevein-type antimicrobial peptide from the bark of spindle tree (*Euonymus europaeus* L.). *FEBS Lett.* 530 181–185. 10.1016/S0014-5793(02)03474-9 12387889

[B53] Van den Bergh K. P. Rouge P. Proost P. Coosemans J. Krouglova T. Engelborghs Y. (2004). Synergistic antifungal activity of two chitin-binding proteins from spindle tree (*Euonymus europaeus* L.). *Planta* 219 221–232. 10.1007/s00425-004-1238-1 15048569

[B54] Van den Bergh K. P. Van Damme E. J. Peumans W. J. Coosemans J. (2002b). Ee-CBP, a hevein-type antimicrobial peptide from bark of the spindle tree (*Euonymus europaeus* L.). *Meded. Rijksuniv. Gent. Fak. Landbouwkd. Toegep. Biol. Wet.* 67 327–331. 12701440

[B55] vanBeek T. A. (2003). *Ginkgo Biloba.* Boca Raton, FL: CRC Press.

[B56] Wiegand I. Hilpert K. Hancock R. E. (2008). Agar and broth dilution methods to determine the minimal inhibitory concentration (MIC) of antimicrobial substances. *Nat. Protoc.* 3 163–175. 10.1038/nprot.2007.521 18274517

[B57] Wilkins M. R. Gasteiger E. Bairoch A. Sanchez J. C. Williams K. L. Appel R. D. (1999). Protein identification and analysis tools in the ExPASy server. *Methods Mol. Biol.* 112 531–552. 10.1385/1-59259-890-0:571 10027275

[B58] Wong K. H. Li G. Q. Li K. M. Razmovski-Naumovski V. Chan K. (2011). Kudzu root: traditional uses and potential medicinal benefits in diabetes and cardiovascular diseases. *J. Ethnopharmacol.* 134 584–607. 10.1016/j.jep.2011.02.001 21315814

[B59] Wong K. H. Razmovski-Naumovski V. Li K. M. Li G. Q. Chan K. (2013). Differentiation of *Pueraria lobata* and *Pueraria thomsonii* using partial least square discriminant analysis (PLS-DA). *J. Pharm. Biomed. Anal.* 84 5–13. 10.1016/j.jpba.2013.05.040 23777642

[B60] Wong K. H. Razmovski-Naumovski V. Li K. M. Li G. Q. Chan K. (2014). Differentiating puerariae lobatae radix and puerariae thomsonii radix using HPTLC coupled with multivariate classification analyses. *J. Pharm. Biomed. Anal.* 95 11–19. 10.1016/j.jpba.2014.02.007 24631955

[B61] Wong K. H. Razmovski-Naumovski V. Li K. M. Li G. Q. Chan K. (2015a). Comparing morphological, chemical and anti-diabetic characteristics of puerariae lobatae radix and puerariae thomsonii radix. *J. Ethnopharmacol.* 164 53–63. 10.1016/j.jep.2014.12.050 25560667

[B62] Wong K. H. Razmovski-Naumovski V. Li K. M. Li G. Q. Chan K. (2015b). The quality control of two *Pueraria* species using raman spectroscopy coupled with partial least squares analysis. *J. Raman Spectrosc.* 46 361–368. 10.1002/jrs.4652

[B63] Ye X. Y. Ng T. B. (2002). A new antifungal peptide from rice beans. *J. Pept. Res.* 60 81–87. 10.1034/j.1399-3011.2002.20962.x 12102720

[B64] Zasloff M. (2002). Antimicrobial peptides of multicellular organisms. *Nature* 415 389–395. 10.1038/415389a 11807545

[B65] Zhang A. L. Story D. F. Lin V. Vitetta L. Xue C. C. (2008). A population survey on the use of 24 common medicinal herbs in Australia. *Pharmacoepidemiol. Drug Saf.* 17 1006–1013. 10.1002/pds.1610 18816875

