# Supplementary material for: Ginkgotides: Proline-Rich Hevein-Like Peptides from Gymnosperm Ginkgo biloba
Source: Front Plant Sci. 2016 Nov 3;7:1639. doi: 10.3389/fpls.2016.01639 (PMC5093130; doi:10.3389/fpls.2016.01639)
Supplement: Supplementary file 1 [file Data_Sheet_1.docx]

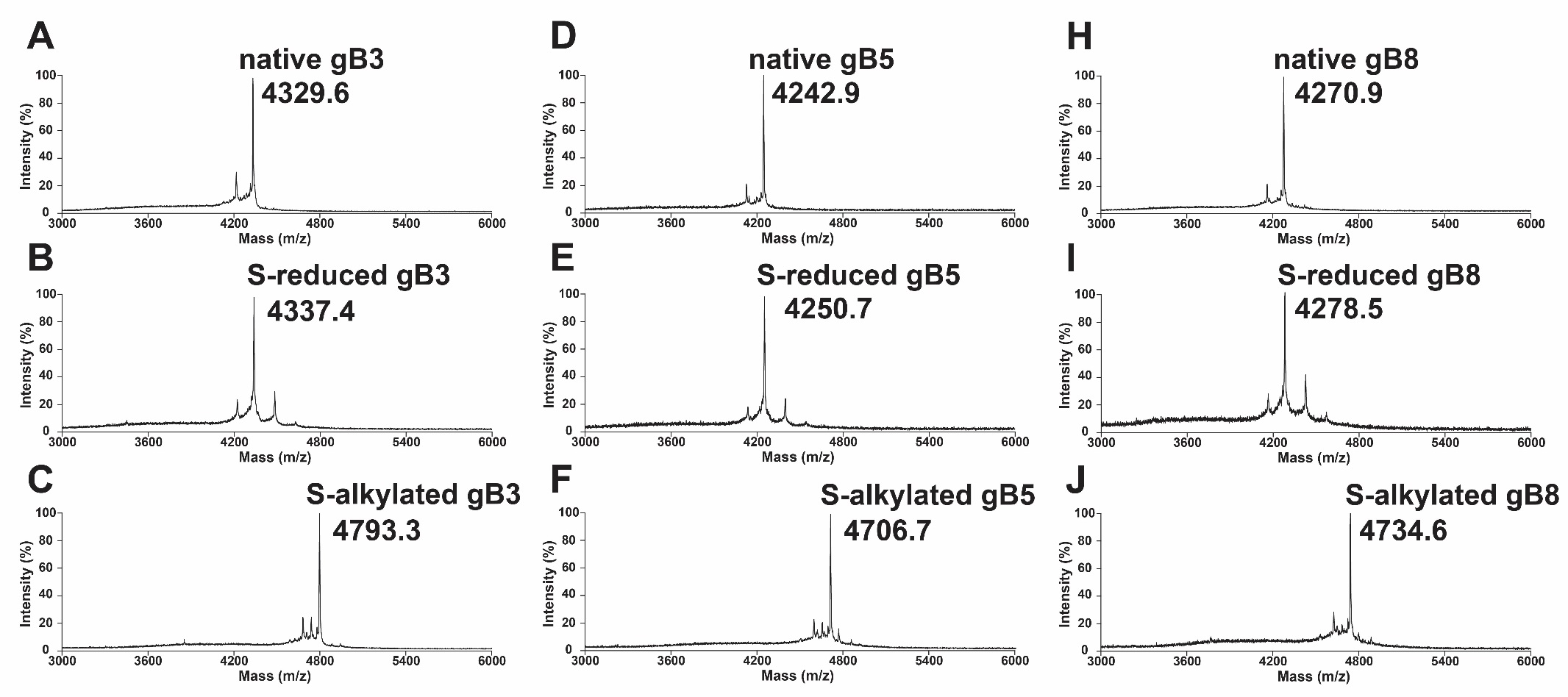


Figure S1. The MALDI-TOF spectra of ginkgotides. The native ginkgotides gB3, gB5 and gB8 (A, D & H) were S-reduced by dithiothreitol (B, E & I) and S-alkylated by iodoacetamide. The mass difference before and after the reductive S-alkylation of ginkgotides was monitored using MALDI-TOF MS. Each S-alkylated Cys caused a mass increase of 58 Da. A mass shift of 464 Da (C, F & J) suggests the presence of eight Cys in each peptide.


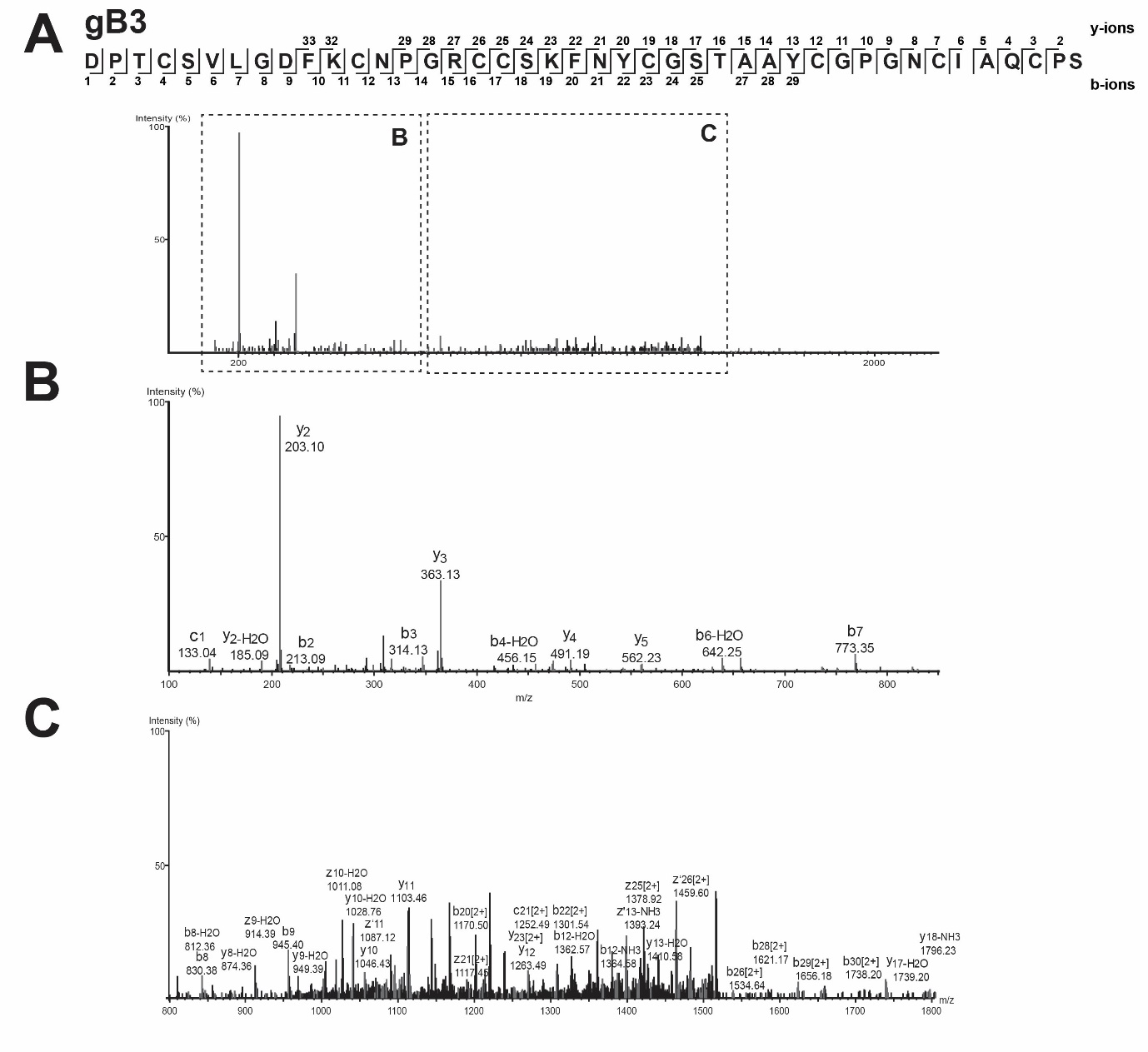


Figure S2. Mass spectra of ginkgotides gB3 from LC-ESI-LTQ-Orbitrap MS/MS in positive ion mode. The purified gB5 was S-reduced by 20 mM dithiothreitol, S-alkylated with 200 mM iodoacetic acid and subsequently de-salted by C_18_ Ziptip. The spectrum was scanned between mass ranges of (A) 100 and 2000 m/z, (B) 100 and 900 m/z and (C) 800 and 1800 m/z. Assignment of isobaric amino acids such as Leu/Ile were confirmed by the transcriptome.


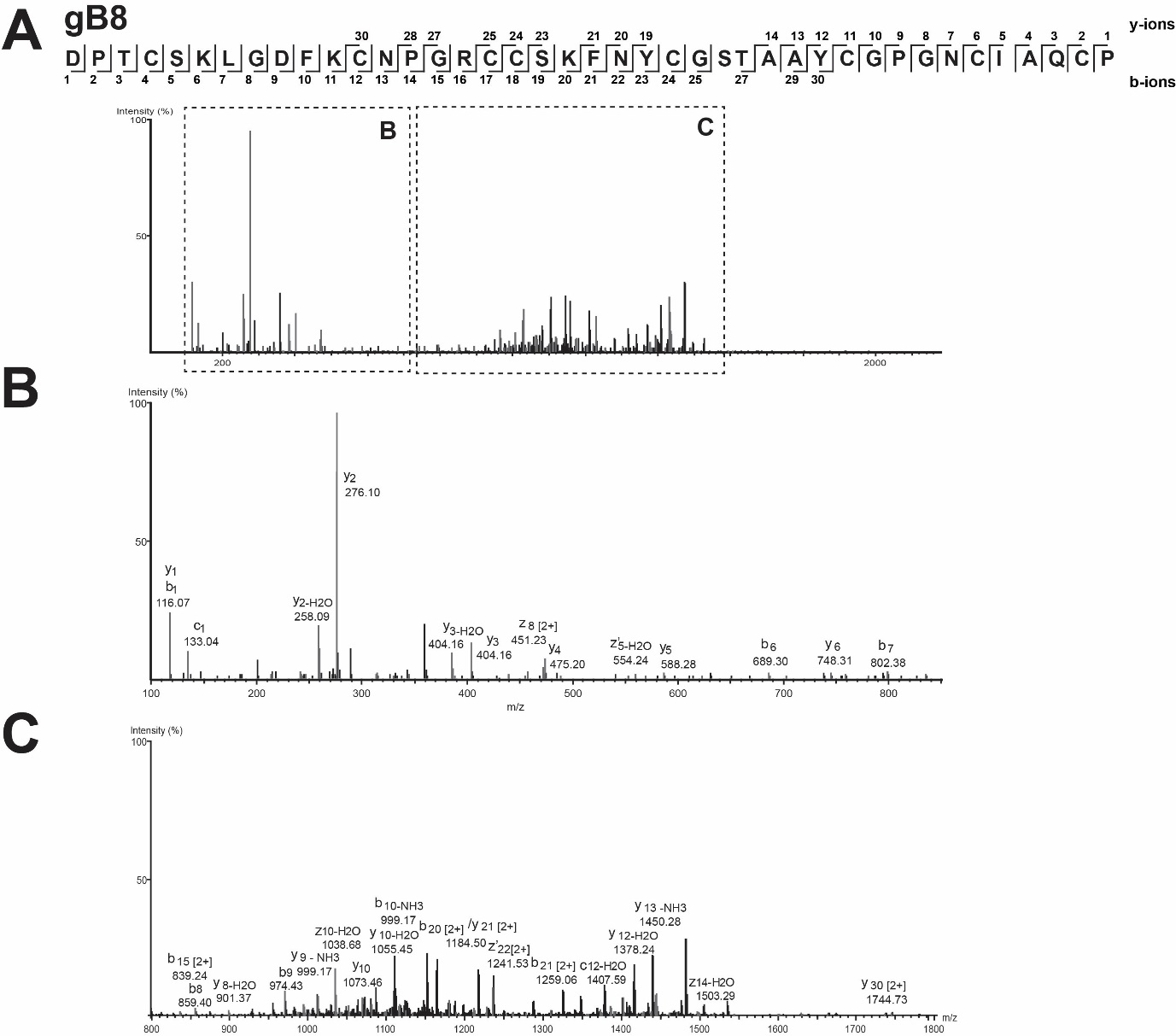


Figure S3. Mass spectra of ginkgotides gB8 from LC-ESI-LTQ-Orbitrap MS/MS in positive ion mode. The purified gB5 was S-reduced by 20 mM dithiothreitol, S-alkylated with 200 mM iodoacetic acid and subsequently de-salted by C_18_ Ziptip. The spectrum was scanned between mass ranges of (A) 100 and 2000 m/z, (B) 100 and 900 m/z and (C) 800 and 1800 m/z. Assignment of isobaric amino acids such as Leu/Ile were confirmed by the transcriptome.


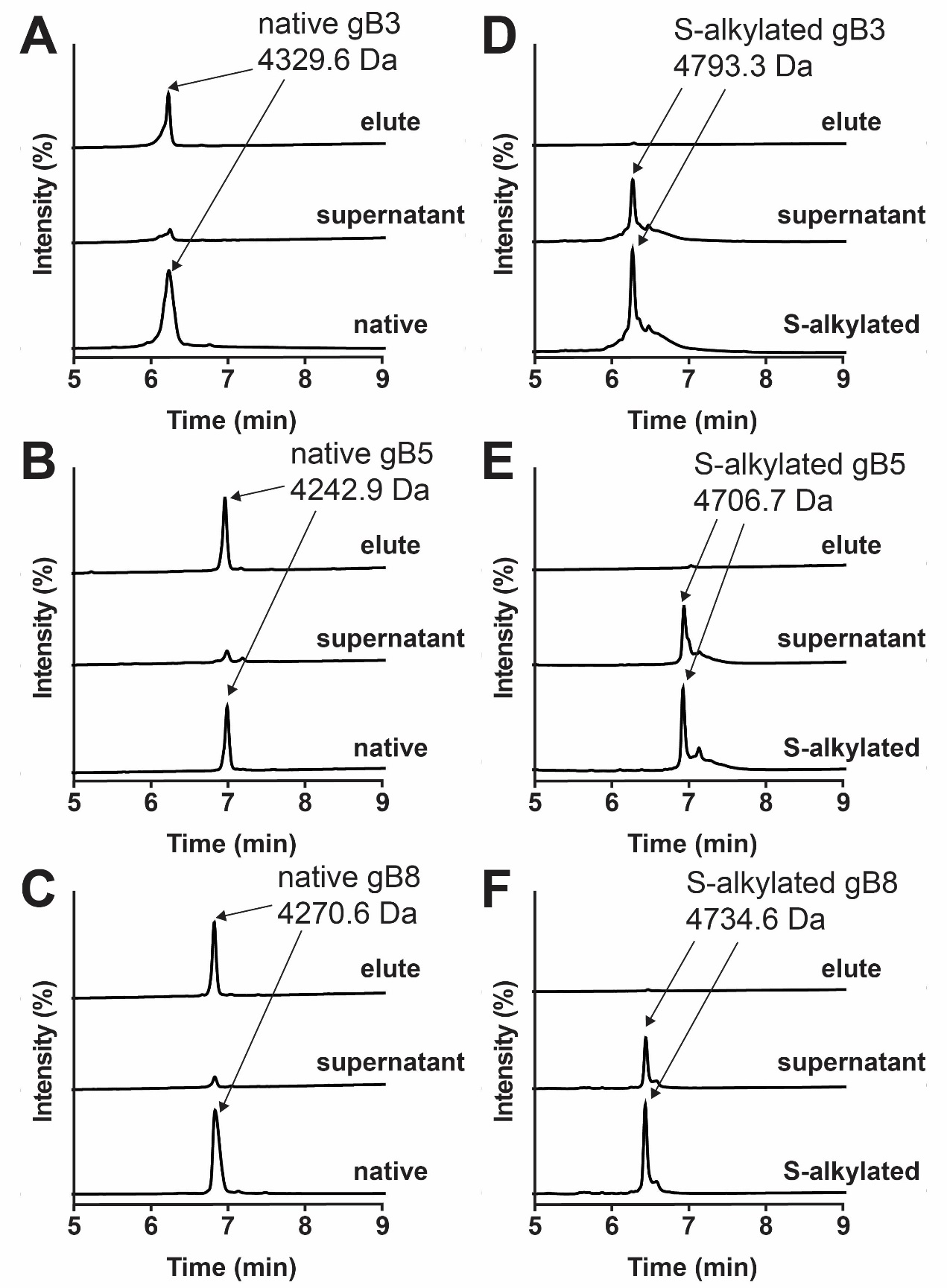


Figure S4. Comparison of the chitin-binding activity between native and S-alkylated ginkgotides. The native (A, B & C) and S-alkylated (D, E & F) ginkgotides gB3, gB5 and gB8 were incubated with chitin beads in buffer (140 mM NaCl, 10 mM Tris, 1 mM EDTA and 0.1% (v/v) Tween at pH 8.0) for 4 hr. Chitin-bound peptides were eluted by 500 mM acetic acid (pH 3.0). The supernatants and eluents were analyzed using UPLC and MALDI-TOF MS.

Figure S5. 2D-NOESY slices of residue C12, K11 and F10. For each residue (*i*), HN had a correlation peak with Hα of the residue before it (*i*-1). The sequential connection was displayed in solid line.


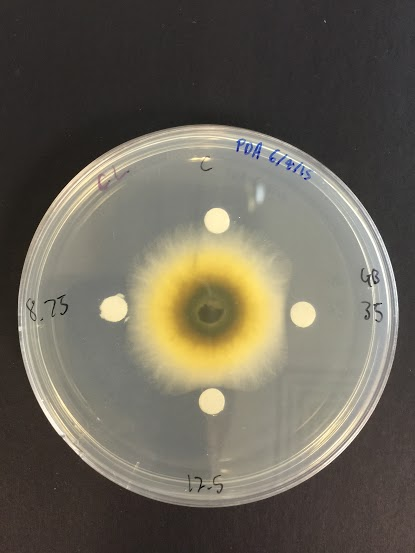

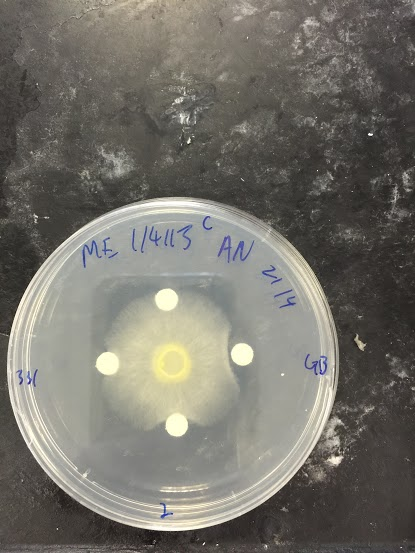

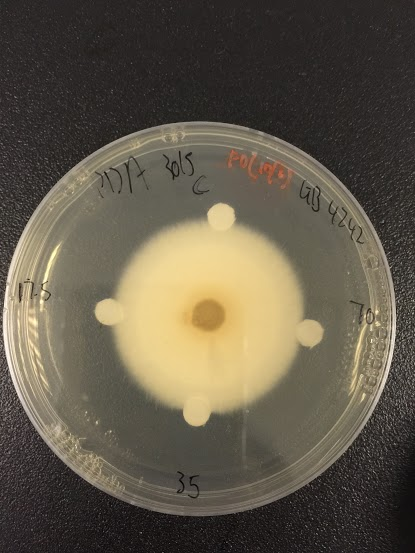

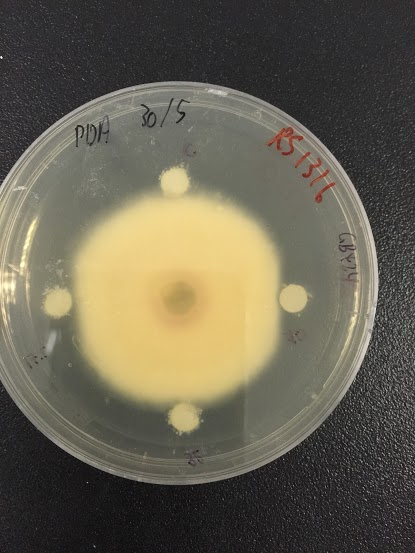


**A**

**B**

**C**

**D**

**1**

**2**

**4**

**3**

**1**

**2**

**4**

**3**

**1**

**2**

**4**

**3**

**1**

**2**

**3**

**4**

Figure S6. Anti-fungal disk diffusion assay of gB5. Crescent-shaped zones were observed around the gB5-treated disk with concentration of (2) 8.75, (3) 12.5 and (3) 35 µg/mL. (1) Milli-Q water was used as the control disk. These results suggested that gB5 exerted an anti-fungal activity against the hyphal growth of (A) *Aspergillus niger*, (B) *Curvularia lunata*, (C) *Fusarium oxysporum* and (D) *Rhizoctonia solani*.

Table S1. NMR experimental and structural statistics of gB5.

| **NMR Distance Restraints** |  |
| --- | --- |
| Intra-Residue NOE (\|i-j\|=0) | 128 |
| Sequential NOE(\|i-j\|=1) | 127 |
| Medium-Range NOE (1<\|i-j\|≤5) | 70 |
| Long-Range NOE (\|i-j\|>5) | 114 |
| All | 439 |
| Hydrogen Bonds | 6 |
| **Structural Statistics (41 residues, D1-P41)** |  |
| NOE Violation | 0.025 ± 0.001 Å |
| Maximum NOE Violation | 0.028 Å |
| Ramachandran Plot Region (41 residues) |  |
| Residues in Most Favored Regions | 21 (67.7%) |
| Residues in Additional Allowed Regions | 7 (22.6%) |
| Residues in Generously Allowed Regions | 2 (9.7%) |
| Residues in Disallowed Regions | 0 (0%) |
| Number of End-Residues (excl. Gly and Pro) | 1 |
| Number of Glycine Residues | 5 |
| Number of Proline Residues | 4 |
| **Mean RMSD from the Average Coordinates (33 residues, D9-P41)** | |
| Backbone Atoms | 0.49 ± 0.17 |
| Heavy Atoms | 0.99 ± 0.20 |

Table S2. The averaged energies of the 20 best structures of gB5.

|  | **Disulfide Bond Pattern** | **Energy (kcal/mol)** |
| --- | --- | --- |
| **1** | CysI-CysIV, CysII-CysV, CysIII-CysVI, CysVII-CysVIII | 546.74 ± 9.169 |
| **2** | CysI-CysIV, CysII-CysV, CysIII-CysVII, CysVI-CysVIII | 566.73 ± 4.372 |
| **3** | CysI-CysIV, CysII-CysV, CysIII-CysVIII, CysVII-CysVI | 577.50 ± 9.721 |
| **4** | CysI-CysIII, CysII-CysV, CysIV-CysVI, CysVII-CysVIII | 872.70 ± 11.639 |
| **5** | CysI-CysIII, CysII-CysV, CysIV-CysVII, CysVI-CysVIII | 891.45 ± 17.131 |
| **6** | CysI-CysIII, CysII-CysV, CysIV-CysVIII, CysVI-CysVII | 912.36 ± 27.135 |
| **7** | CysI-CysVI, CysII-CysV, CysIV-CysVII, CysIII-CysVIII | 1030.89 ± 9.580 |
| **8** | CysI-CysVI, CysII-CysV, CysIV-CysVIII, CysIII-CysVII | 1089.02 ± 71.898 |
| **9** | CysI-CysVII, CysII-CysV, CysIV-CysVI, CysIII-CysVIII | 1092.81 ± 25.890 |
| **10** | CysI-CysVII, CysII-CysV, CysIV-CysVIII, CysIII-CysVI | 1018.83 ± 17.652 |
| **11** | CysI-CysVIII, CysII-CysV, CysIV-CysVI, CysIII-CysVII | 977.96 ± 16.858 |
| **12** | CysI-CysVIII, CysII-CysV, CysIV-CysVII, CysIII-CysVI | 1442.84 ± 111.916 |
| **13** | CysI-CysVI, CysII-CysV, CysIII-CysIV, CysVII-CysVIII | 1079.64 ± 12.375 |
| **14** | CysI-CysVII, CysII-CysV, CysIII-CysIV, CysVI-CysVIII | 1086.85 ± 13.276 |
| **15** | CysI-CysVIII, CysII-CysV, CysIII-CysIV, CysVI-CysVII | 937.58 ± 38.311 |

Table S3. NMR atom chemical shift list of gB5.

| **Residue** | **HN** | **CH_α_** |  | **CH_β_** |  | **Others** |  |  |  |
| --- | --- | --- | --- | --- | --- | --- | --- | --- | --- |
| **P2** |  | 5.021 |  | 2.294 | 2.12 | H_δ_ 3.694 | H_ϒ_ 1.914 |  |  |
| **T3** | 8.033 | 4.794 |  | 4.198 |  | H_ϒ_ 0.967 |  |  |  |
| **C4** | 7.393 | 4.581 |  | 3.088 | 2.902 |  |  |  |  |
| **S5** | 8.06 | 4.502 |  | 4.02 | 3.824 |  |  |  |  |
| **V6** | 8.639 | 3.812 |  | 2.097 |  | H_ϒ_ 1.008, 0.976 |  |  |  |
| **L7** | 8.132 | 4.364 |  | 1.65 |  | H_ϒ_ 1.567 | H_δ_ 0.891, 0.859 |  |  |
| **G8** | 7.902 | 3.925 | 3.805 |  |  |  |  |  |  |
| **D9** | 8.251 | 4.274 |  | 2.576 | 2.334 |  |  |  |  |
| **F10** | 7.548 | 4.616 |  | 2.83 | 2.523 | H_δ_ 7.072 |  |  |  |
| **K11** | 8.813 | 4.827 |  | 1.85 | 1.7 | H_δ_ 1.58 | H_ε_ 2.957 | H_ϒ_ 1.329 | H_ζ_ 7.317 |
| **C12** | 9.796 | 5.023 |  | 2.89 | 2.38 |  |  |  |  |
| **N13** | 10.422 | 4.733 |  | 2.928 | 2.371 | H_δ_ 7.558, 7.08 |  |  |  |
| **P14** |  | 4.23 |  | 2.277 | 2.106 | H_δ_ 3.827, 3.506 | H_ϒ_ 1.799 |  |  |
| **G15** | 8.687 | 4.232 | 3.605 |  |  |  |  |  |  |
| **R16** | 8.028 | 4.415 |  | 1.816 | 1.567 | H_δ_ 2.632 | H_ε_ 9.498 | H_ϒ_ 1.326, 1.113 |  |
| **C17** | 9.1 | 4.824 |  | 4.253 | 2.326 |  |  |  |  |
| **C18** | 8.245 | 5.018 |  | 2.956 | 2.788 |  |  |  |  |
| **S19** | 9.447 | 5.317 |  | 4.202 |  |  |  |  |  |
| **K20** | 8.186 | 4.067 |  | 1.726 |  | H_δ_ 1.489 | H_ϒ_ 1.15 | H_ζ_ 7.407 |  |
| **F21** | 7.71 | 4.823 |  | 3.442 | 2.929 | H_δ_ 7.259, 7.199 |  |  |  |
| **N22** | 8.578 | 4.163 |  | 3.439 | 3.003 | H_δ_ 7.99 |  |  |  |
| **Y23** | 7.236 | 5.4 |  | 3.393 | 2.814 | H_δ_ 7.199, 6.698 |  |  |  |
| **C24** | 8.25 | 5.737 |  | 2.667 |  |  |  |  |  |
| **G25** | 8.304 | 3.532 | 2.031 |  |  |  |  |  |  |
| **S26** | 9.127 | 5.193 |  | 4.029 | 3.756 |  |  |  |  |
| **T27** | 7.078 | 4.847 |  | 4.694 |  | H_ϒ_ 1.37 |  |  |  |
| **A28** | 9.183 | 4.1 |  | 1.498 |  |  |  |  |  |
| **A29** | 7.865 | 3.966 |  | 1.153 |  |  |  |  |  |
| **Y30** | 7.763 | 4.126 |  | 2.997 | 2.797 | H_δ_ 7.339 |  |  |  |
| **C31** | 7.903 | 4.521 |  | 3.114 | 2.589 |  |  |  |  |
| **G32** | 8.399 | 4.319 | 3.929 |  |  |  |  |  |  |
| **P33** |  | 4.38 |  | 2.275 | 2.113 | H_δ_ 3.797, 3.59 | H_ϒ_ 2.041, 1.92 |  |  |
| **G34** | 8.881 | 4.178 | 3.641 |  |  |  |  |  |  |
| **N35** | 7.931 | 4.736 |  | 2.861 | 2.346 | H_δ_ 6.731, 7.175 |  |  |  |
| **C36** | 7.373 | 4.804 |  | 3.39 | 2.763 |  |  |  |  |
| **I37** | 9.497 | 4.633 |  | 1.876 |  | H_δ_ 0.725 | H_ϒ1_ 0.855 | H_ϒ2_ 0.8 |  |
| **A38** | 7.984 | 4.451 |  | 1.432 |  |  |  |  |  |
| **Q39** | 8.979 | 3.816 |  | 2.209 | 2.029 | H_δ_ 7.641 | H_ϒ_ 2.431, 2.383 |  |  |
| **C40** | 7.972 | 5.03 |  | 3.439 | 2.575 |  |  |  |  |
| **P41** |  | 4.326 |  | 2.279 | 2.039 | H_δ_ 3.768 | H_ϒ_1.959, 1.915 |  |  |
| **P2** |  | 5.021 |  | 2.294 | 2.12 | H_δ_ 3.694 | H_ϒ_ 1.914 |  |  |
| **T3** | 8.033 | 4.794 |  | 4.198 |  | H_ϒ_ 0.967 |  |  |  |
| **C4** | 7.393 | 4.581 |  | 3.088 | 2.902 |  |  |  |  |
| **S5** | 8.06 | 4.502 |  | 4.02 | 3.824 |  |  |  |  |
| **V6** | 8.639 | 3.812 |  | 2.097 |  | H_ϒ_ 1.008, 0.976 |  |  |  |
| **L7** | 8.132 | 4.364 |  | 1.65 |  | H_ϒ_ 1.567 | H_δ_ 0.891, 0.859 |  |  |
| **G8** | 7.902 | 3.925 | 3.805 |  |  |  |  |  |  |
| **D9** | 8.251 | 4.274 |  | 2.576 | 2.334 |  |  |  |  |
| **F10** | 7.548 | 4.616 |  | 2.83 | 2.523 | H_δ_ 7.072 |  |  |  |
